# Calcium‐dependent antimicrobials: Nature‐inspired materials and designs

**DOI:** 10.1002/EXP.20230099

**Published:** 2024-03-12

**Authors:** Zhong Wang, Yongjie Zeng, Zubair Ahmed, Hui Qin, Ijaz Ahmad Bhatti, Huiliang Cao

**Affiliations:** ^1^ Interfacial Electrochemistry and Biomaterials School of Materials Science and Engineering East China University of Science and Technology Shanghai China; ^2^ Department of Orthopaedics Shanghai Jiaotong University Affiliated Sixth People's Hospital Shanghai China; ^3^ Department of Chemistry University of Agriculture Faisalabad Pakistan; ^4^ Engineering Research Center for Biomedical Materials of Ministry of Education East China University of Science and Technology Shanghai China; ^5^ Key Laboratory for Ultrafine Materials of Ministry of Education East China University of Science & Technology Shanghai China

**Keywords:** antibacterial materials, implantable medical devices, infections, microbe, osseointegration

## Abstract

Bacterial infection remains a major complication answering for the failures of various implantable medical devices. Tremendous extraordinary advances have been published in the design and synthesis of antimicrobial materials addressing this issue; however, the clinical translation has largely been blocked due to the challenge of balancing the efficacy and safety of these materials. Here, calcium's biochemical features, natural roles in pathogens and the immune systems, and advanced uses in infection medications are illuminated, showing calcium is a promising target for developing implantable devices with less infection tendency. The paper gives a historical overview of biomedical uses of calcium and summarizes calcium's merits in coordination, hydration, ionization, and stereochemistry for acting as a structural former or trigger in biological systems. It focuses on the involvement of calcium in pathogens’ integrity, motility, and metabolism maintenance, outlining the potential antimicrobial targets for calcium. It addresses calcium's uses in the immune systems that the authors can learn from for antimicrobial synthesis. Additionally, the advances in calcium's uses in infection medications are highlighted to sketch the future directions for developing implantable antimicrobial materials. In conclusion, calcium is at the nexus of antimicrobial defense, and future works on taking advantage of calcium in antimicrobial developments are promising in clinical translation.

## Graphical abstract

The paper summarizes calcium's merits in coordination, hydration, ionization, and stereochemistry for acting as a structural former or trigger in biological systems, illuminates its natural roles in pathogens and the immune systems, and discusses its applications in infection medications. It shows that calcium is at the nexus of antimicrobial defense but is overlooked to some extent. Future works on developing calcium‐based antimicrobial strategies are promising in clinical translation.

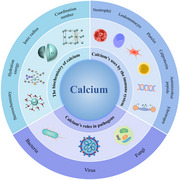

## 1 INTRODUCTION

Medical devices, such as wound dressings, artificial joints, and dental implants, are acknowledged as effective and irreplaceable cures for replacing and repairing damaged human tissue and restoring patients’ functions.^[^ ^1^, ^2^ ^]^ Innumerable wound dressings are consumed every day to treat different kinds of wounds, including surgical sites, burns, and diabetic ulcers.^[^ ^3^ ^]^ The projected demand for total hip and total knee arthroplasties is expected to reach 572,000 and 3.48 million respectively by 2030, representing a growth of 137% and 601% compared to the figures in 2005.^[^ ^4^ ^]^ Every year, approximately 10 million individuals require tooth replacement therapies.^[^ ^5^ ^]^ Unfortunately, bacterial infections remain the leading obstacle to the success of these devices.^[^ ^6^, ^7^ ^]^ The incidence of infections for dental implants was 4–10% within the first three years after insertion, answering two‐thirds of the failure cases.^[^ ^8^ ^]^ Although the infection rates for orthopedic implants are relatively low, for example, 0.5–5% for total joint replacements, the total number of infection cases (accompanied by a mortality rate of about 2.5%) is big due to the increasing large patient population expected.^[^ ^9^, ^10^ ^]^ Device‐associated infections (DAIs) largely delay the recovery of patients and impose a significant financial burden on the worldwide healthcare system. The treatment expense for infected patients was about 6.5 times higher than those for uninfected cases, costing more than $8.6 billion in the United States annually.^[^ ^11^, ^12^ ^]^ Despite the current unsatisfactory failure rate,^[^ ^13^, ^14^ ^]^ hospital DAI prevention and treatment still rely on various antibiotics.^[^ ^15^ ^]^ The heavy antibiotic use produces increasing numbers of resistant pathogens and in turn boosts up the failures of antibiotic therapies, making medical device placements the hardest hit.^[^ ^16^ ^]^

This situation attracted tremendous efforts from academic communities worldwide, producing over 420,000 papers on the ‘*antibacterial*’ topic during the past decade (Web of Science, https://www.webofscience.com/wos/alldb/basic‐search). A vast number of materials have been developed for the fight against bacterial colonization;^[^ ^17^ ^]^ however, most of these strategies can hardly satisfy simultaneously the primary functions and the disinfection needs of a medical device, hindering their clinical translation.^[^ ^18^, ^19^ ^]^ Calcium, the most abundant metal in humans, serves as a trigger and regulator for the innate immune system and tissue healing processes.^[^ ^20^, ^21^, ^22^, ^23^ ^]^ It is demonstrated that calcium involves entirely the neutrophil‐mediated immune process, from cell recruitment and infiltration to the production of antibacterial substances and bacterial phagocytosis.^[^ ^24^ ^]^ The bactericidal strength of natural human antimicrobial peptides, namely defensin and cathelicidin families predominantly produced by neutrophils, leukomonocytes, or cutaneous epithelial cells, is closely dependent on the presence and binding of calcium.^[^ ^25^, ^26^, ^27^, ^28^ ^]^ In addition, calcium interacts directly with several immune and pro‐healing components, that is, platelets, and fibrin(ogen) to reinforce their tissue repair or disinfection efficacies.^[^ ^29^, ^30^, ^31^ ^]^ Previously, improved bone formation and gingival sealing around titanium were reported after calcium doping via ion implantation or hydrothermal treatment.^[^ ^32^, ^33^ ^]^ Our study demonstrated that calcium‐doped titanium has limited in vitro activity against bacterial colonization;^[^ ^34^ ^]^ however, recently we found that calcium‐doped titanium is capable of guiding the adsorption of fibrinogen to prevent bacterial adhesion in vitro.^[^ ^35^ ^]^ Given the unexpected bone integration property of calcium‐doped titanium even under the challenge of resistant bacterial species,^[^ ^34^ ^]^ we believe that calcium is a promising target that can be taken advantage of to balance the primary functions and the disinfection needs of various medical devices, which are highly desired for clinical uses.

Nevertheless, no topical review on the antibacterial roles of calcium has yet been published. Here, we address calcium's biochemical features, natural roles in pathogens and the immune systems, and advanced uses in infection medications to establish an integrated view of calcium in antimicrobial defense. We present the ideas in five steps. After giving a historical overview of calcium's biomedical uses, we illuminate calcium's biochemical features for acting as structural former or trigger in proteins. Then, we address calcium's natural uses in pathogens and the immune systems, and the advanced applications of calcium in infection medications. Finally, we conclude that calcium is a promising target in antimicrobial defense and future works on developing calcium‐based antimicrobials have good prospects in clinical translation.

## 2 A HISTORICAL OVERVIEW OF BIOMEDICAL CALCIUM

Pure calcium was identified and isolated in the early 19th century;^[^ ^36^ ^]^ however, it was not until 1894, that calcium was demonstrated indispensable for the generation and transmission of nerve excitations, eliciting all types of muscle contraction.^[^ ^37^ ^]^ Subsequent studies in the early 20th century found that appropriate calcium deficiency possibly decreased the respiratory rate in humans,^[^ ^38^ ^]^ this possibly laid the foundation for using calcium supplements to mitigate the side effects of the Coronavirus Disease 2019 (COVID‐19) in severe patients with hypocalcemia (a serum calcium concentration below the normal range).^[^ ^39^ ^]^ In 1934, the reversible binding between calcium ions and serum proteins was first reported in a frog heart model.^[^ ^40^ ^]^ Later in 1942, calcium was found acting as a potent activator for myosin ATPase, controlling the ATPase's enzymatic activity.^[^ ^41^ ^]^ These findings subsequently promoted the development of calcium antagonists (coined in 1967) for inhibiting calcium access through voltage‐activated ion channels to manage various diseases, especially cardiac disorders.^[^ ^42^, ^43^, ^44^ ^]^ In 1980, calmodulin was initially introduced as a calcium‐specific binding protein, whose pivotal roles in diverse cellular behaviors and activities were evidenced in subsequent studies.^[^ ^45^, ^46^ ^]^ In 1984, intracellular calcium was recognized as a ubiquitous regulator,^[^ ^47^ ^]^ and since 1985, calcium has been known to act as a second messenger in a wide range of inositol trisphosphate triggered functions in mammalian cells.^[^ ^48^ ^]^ This biological evidence promoted the application of various calcium compounds for promoting tissue healing. Calcium chloride was first used to achieve hemostasis for surgeries in 1896,^[^ ^49^ ^]^ soon afterward calcium was known as a crucial factor in enzyme activation and coagulation.^[^ ^50^ ^]^ The ‘triple calcium phosphate’ as a stimulus to osteogenesis was first validated by Albee and Morrison in 1920.^[^ ^51^ ^]^ They found that bone growth and union of the fractures in rabbits was significantly accelerated by injection of a ‘triple calcium phosphate’ solution (5%). Later, Blaine introduced calcium chloride into alginate to produce calcium alginate and initiate clotting for promoted bone, muscle, skin, and sclera healing.^[^ ^52^ ^]^ Monroe et al. fabricated a porous hydroxyapatite ceramic via sintering the mixture of fine‐grained synthetic hydroxyapatite (known as basic calcium phosphate) particles and colloidal cellulose, which was able to promote osseointegration of a bone or tooth implant.^[^ ^53^ ^]^ Other formulas, such as calcium peroxide, calcium fluoride, sodium calcium borate glass, and calcium hydride, were also found good for tissue regeneration.^[^ ^54^, ^55^, ^56^, ^57^ ^]^ Moreover, some studies demonstrated the side effects of calcium on tissue healing. In 1984, Poole‐Wilson et al. proposed calcium is invasive in the pathogenesis of myocardial cell necrosis following severe hypoxia or ischemia, acting in three different ways toward cell damage, including depletion of energy and loss of the enzyme system, disruption of the cell matrix, and disintegration of plasma and mitochondrial membranes.^[^ ^58^ ^]^

**FIGURE 1:**
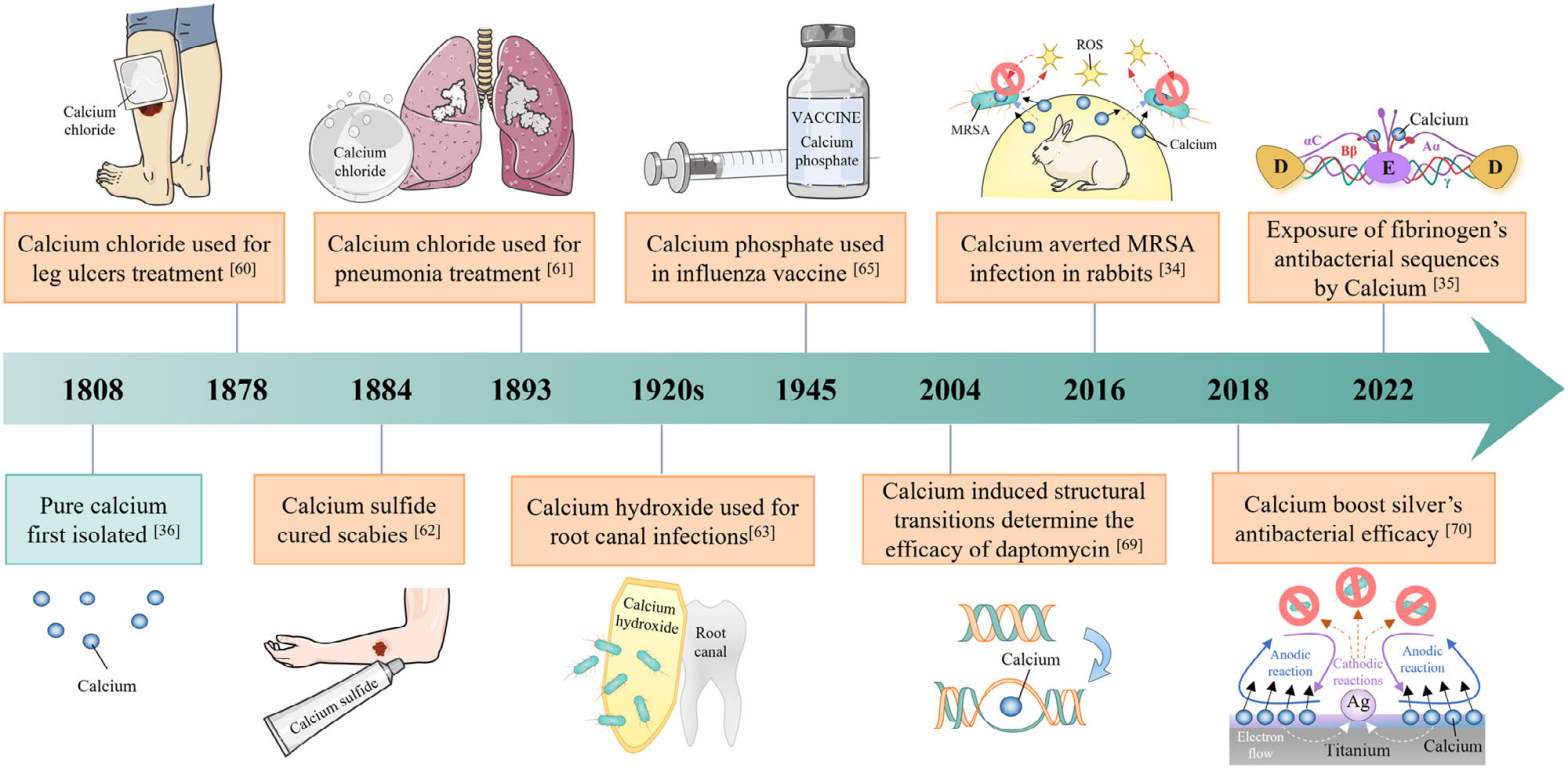
Calcium's milestones in antimicrobial defense. Methicillin‐resistant *Staphylococcus aureus* (MRSA); Reactive oxygen species (ROS); Silver (Ag). The illustration of 2018 was adapted with permission.^[^ ^70^ ^]^ Copyright 2018, The Royal Society of Chemistry. The illustration of 2022 was adapted with permission.^[^ ^35^ ^]^ Copyright 2022, The Royal Society of Chemistry. Other illustrations were produced by the authors.

In addition to uncovering the biological functions of calcium, calcium's uses in antimicrobial defense are relatively limited (Figure 1). It was said that the ancient Egyptians employed calcium sulfate (gypsum) mortar to protect the hull wood from water, oxygen, and bacteria.^[^ ^59^ ^]^ In 1878, calcium chloride‐loaded dressings were found effective in the treatment of leg ulcers.^[^ ^60^ ^]^ Calcium chloride was demonstrated as an effective treatment for pneumonia in 1893.^[^ ^61^ ^]^ Besides, calcium sulfide was identified as an effective cure for scabies in 1884.^[^ ^62^ ^]^ Calcium hydroxide became a preferred filling material for root canal therapies in the 1920s,^[^ ^63^ ^]^ In 1960 this material was demonstrated effective in removing bacterial colonies from periapical tissue by a root canal infection model in a dog.^[^ ^64^ ^]^ Furthermore, calcium phosphate was determined in 1945 as a primary sediment to influenza virus and a good adjuvant for associated vaccines.^[^ ^65^ ^]^ In the 1980s, calcium chloride was found an excellent reinforcer to butylated hydroxyanisole (a prevalent food additive) against *Staphylococcus aureus* colonization.^[^ ^66^, ^67^ ^]^ The reproduction of *Monilinia fructicola* was greatly inhibited by 65% when sprayed with calcium hydroxide, calcium oxide, calcium silicate, or calcium pyrophosphate, demonstrating good efficacy in the reduction of brown rot incidence in fruit plants.^[^ ^68^ ^]^ Although these reports were not implantable device‐associated, they are strong evidence for developing calcium‐based antimicrobials targeting DAIs. Previous studies demonstrated that calcium can boost the efficacy of synthetic antibacterial materials (such as antibiotics^[^ ^69^ ^]^ and nanosilver^[^ ^70^ ^]^) or assist the immune system in bacterial clearance.^[^ ^34^ ^]^ Very recently, our group found that calcium can guide the adsorption of human fibrinogen to titanium, to expose the protein's antimicrobial sequences against the adhesion and growth of *Pseudomonas aeruginosa* (*P. aeruginosa*).^[^ ^35^ ^]^ We are aware that calcium may be a good target for developing implantable medical devices with antibacterial activities, which inspired the present paper.

## 3 THE BIOCHEMISTRY OF CALCIUM

### 3.1 The chemical characteristics of calcium

**FIGURE 2:**
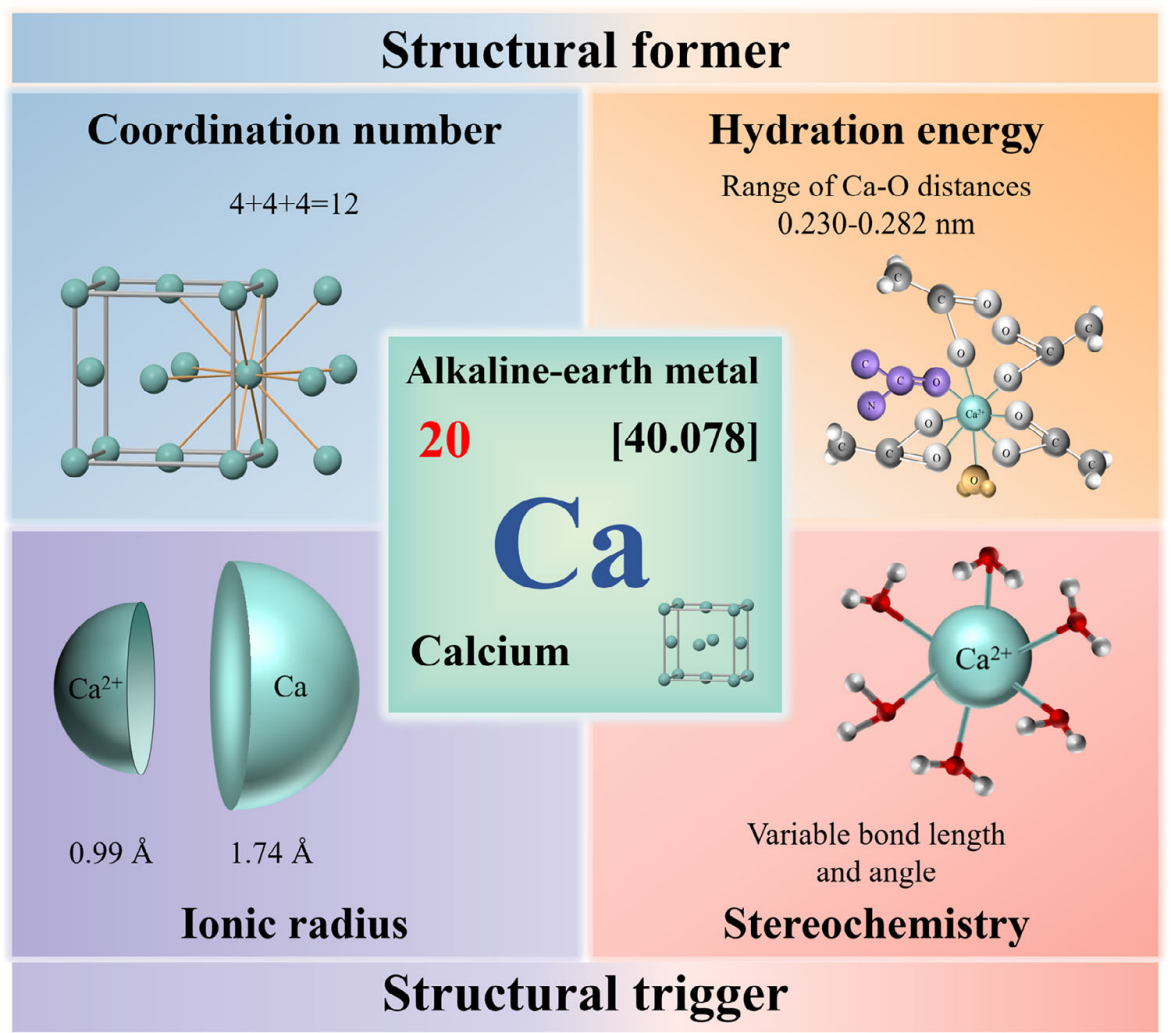
The merits of calcium in living systems: The coordination and hydration features of calcium enable its structural former roles. The ionic radius and stereochemistry characteristics of calcium facilitate its structural trigger actions. The illustration of hydration was adapted with permission.^[^ ^76^ ^]^ Copyright 2016, American Society for Biochemistry and Molecular Biology. The stereochemistry illustration was adapted with permission.^[^ ^79^ ^]^ Copyright 2020, The Royal Society of Chemistry.

Calcium (Ca, atomic number 20) has an atomic weight of 40.078 g mol^−1^ and a density of 1.55 g cm^−3^.^[^ ^71^ ^]^ Its ions can diffuse easily and remain bound for a relatively long time, making them a valuable control in triggering biochemical reactions.^[^ ^72^, ^73^ ^]^ Calcium is also capable of forming complexes with organic compounds or binding to inorganic molecules of low molecular weight.^[^ ^74^ ^]^ In living systems, the merits of calcium are mainly featured in the following aspects (Figure 2): (I) Calcium has high and variable coordination numbers typically ranging from 6 to 8, even up to 12, providing sufficient binding sites for various ligands to form compounds with high structural stability.^[^ ^75^ ^]^ (II) Calcium has a low hydration energy and a small hydrated ion radius (410 kcal g^−1^, 4.5 Å), hence protein does not require a significant conformation change during the attainment of complete six bonds to calcium ions.^[^ ^48^, ^74^, ^76^ ^]^ As a result, calcium's binding efficiency and speed are high, surpassing other divalent ions (e.g. magnesium).^[^ ^48^, ^74^, ^77^ ^]^ (III) Calcium has a small ionic radius (0.99 Å), facilitating its tight binding to proteins,^[^ ^73^ ^]^ especially those with ligands of lower charge density, for example, calmodulin.^[^ ^48^, ^73^ ^]^ (IV) Calcium has variable bond length and angle to ligands, allowing strong multidentate binding without steric hindrance.^[^ ^77^, ^78^, ^79^ ^]^ This unique stereochemistry of calcium proved crucial for the opening and closing of cytomembrane channels or pumps.^[^ ^80^ ^]^ These explain calcium's superiority in crucial signaling of both intracellular and extracellular events.

### 3.2 Calcium's biochemical roles

Calcium can bind rapidly and strongly to organics while allowing quick disassociation when it is necessary, guaranteeing its structural former or trigger roles in biological systems.^[^ ^73^ ^]^ The binding affinity of calcium to proteins is dramatically high to 10^6^–10^7^.^[^ ^81^ ^]^ Calcium can permeate all types of cellular membranes,^[^ ^82^ ^]^ whereas its concentration on either side of the membrane is sophisticatedly regulated and maintained at approximate levels,^[^ ^83^ ^]^ keeping the calcium gradient across cellular membrane range from 10^−2^ to 10^−8^ m.^[^ ^72^ ^]^ Calcium is actively expelled by cells and only utilized by the structural framework in most living systems.^[^ ^82^ ^]^ Its strong binding to cells’ anionic surfaces provides significant strength to the cell membrane or wall.^[^ ^84^ ^]^ At a rest state, the prokaryotes’ calcium flow exhibits steady and localized features which further induces fluctuations in its complex arrangement of pumps, channels, and storage organelles.^[^ ^85^ ^]^ The calcium flow is intricately linked to phosphate metabolism‐associated pumps and enzymes, which in turn affect the transport of other ions (such as hydrogen, sodium, and potassium) and the maintenance of energy status in cells.^[^ ^72^, ^86^, ^87^ ^]^ These processes start with the calcium's binding to specific receptors on cells’ membranes and the recognition by various channels or pumps,^[^ ^86^, ^87^ ^]^ which are determined by calcium's binding strength, unique stereochemistry (variable bond length and angle of ligands), and transmembrane concentration ratios.^[^ ^73^, ^82^, ^88^ ^]^ The primary binding site for calcium is oxygen‐donor centers, which can be either neutral or charged in water, alcohols, ethers, carboxylates, and phosphates.^[^ ^84^ ^]^ As a result, the calcium‐binding sites in proteins (e.g. calmodulin) normally consist of carbonyl groups (from the protein's backbone) and carboxylate groups (from the side chains).^[^ ^89^ ^]^ The binding constant of calcium in calmodulin is calculated to be high up to l0^6^ (calcium's triggered diffusion limit was 10^9^–10^10^ s^−1^ and the off‐rate was 10^3^ s^−1^), demonstrating that calcium is the most suitable substance for the structural trigger.^[^ ^90^ ^]^ The entry of calcium into the cytoplasm can trigger either physical (mechanical) cascades or chemical cascades, often involving phosphorylation and subsequent metabolic alterations. This process also can be categorized into several types based on calcium‐induced reaction time, mechanical cascades (twitch) are the fast calcium response mechanism, a twitch can be completed in less than 10^−2^ s, while the rate of calcium triggering is of the order of 10^−4^ s.^[^ ^72^ ^]^ For example, calcium binds to troponin to trigger a structural change in tropomyosin and expose the binding sites on the actin (thin filaments) for myosin (thick filaments), as a result inducing muscle contractions.^[^ ^91^ ^]^ In this process, calcium acts as a structural trigger.

## 4 CALCIUM'S ROLES IN PATHOGENS

### 4.1 Calcium in bacteria

**FIGURE 3:**
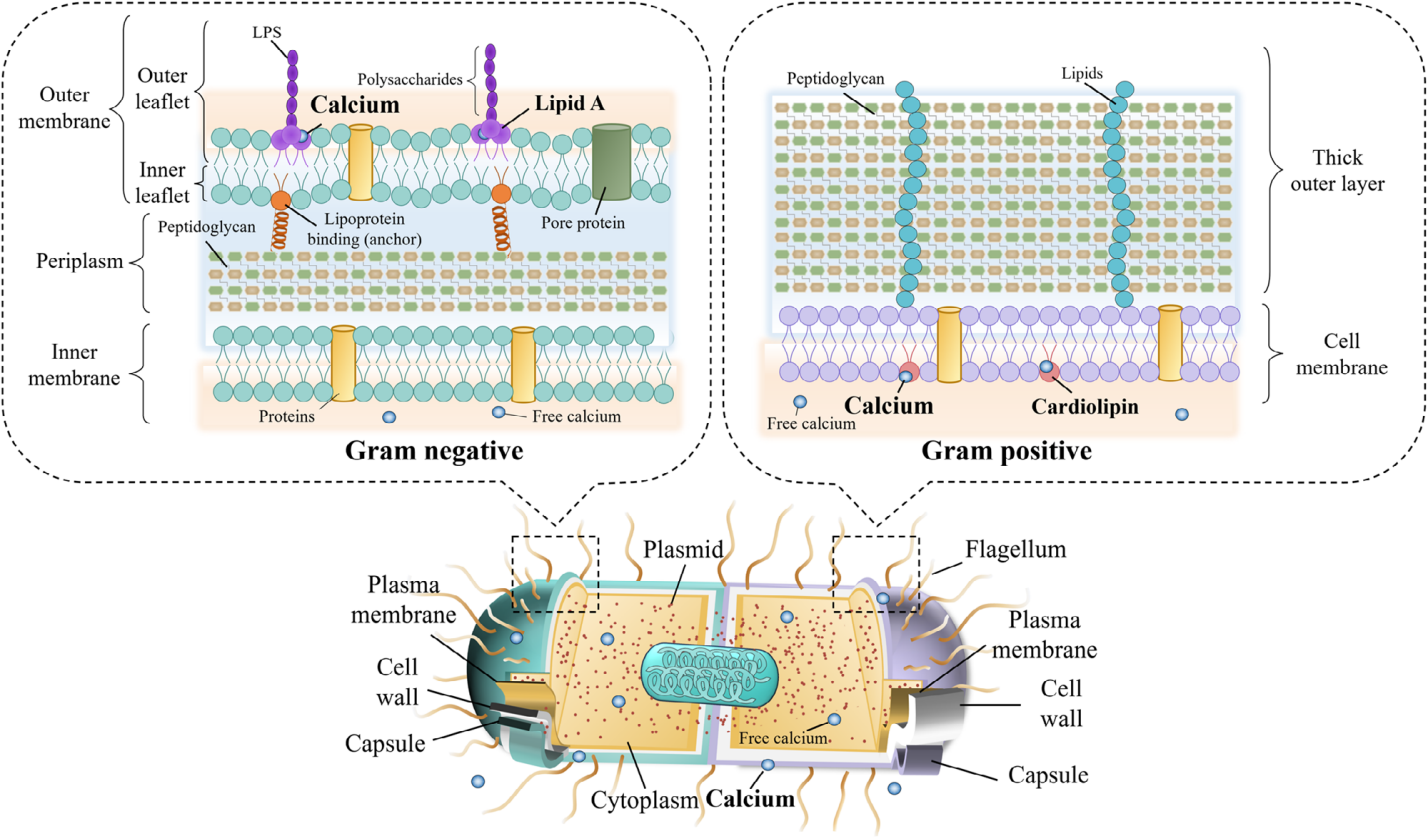
Calcium is crucial for maintaining the integrity of a bacterial envelope. Calcium normally binds to lipids A in Gram‐negative bacteria and cardiolipin in Gram‐positive bacteria, serving as a structural former to stabilize the bacterial envelopes. Lipopolysaccharides (LPS). Adapted with permission.^[^ ^98^ ^]^ Copyright 2021, MDPI.

Calcium is essential for bacterial maintenance, involving cell integrity, motility, and metabolism.^[^ ^92^, ^93^, ^94^, ^95^ ^]^ The binding of extracellular calcium to bacterial surfaces normally stabilizes bacterial membranes, inhibits biofilm dispersion, and serves as a vital source for cytoplasm and extracellular fluid.^[^ ^96^, ^97^ ^]^ Despite the structural composition of Gram‐negative and Gram‐positive membranes exhibiting significant differences, calcium still serves as an essential component for maintaining structural integrity (Figure 3).^[^ ^98^ ^]^ Positive‐charged calcium ions were found to tend to facilitate bacterial aggregation via neutralization of the bacterial surfaces, which are normally negatively charged.^[^ ^99^ ^]^ In addition, the outer surface monolayers in Gram‐negative bacterial cells consist of acidic bilobed proteins arranged in crystalline arrays, in which binding of calcium is involved.^[^ ^100^ ^]^ The outer leaflet of Gram‐negative bacteria's outer membrane is formed by lipopolysaccharides comprising lipids A and polysaccharides.^[^ ^101^ ^]^ High‐affinity binding sites for calcium were detected in the outer lipopolysaccharide layer of *Salmonella typhimurium* (Gram‐negative bacteria).^[^ ^102^ ^]^ Hence, the cell wall integrity of Gram‐negative bacteria such as Filamentous cyanobacteria *Nostoc* PCC 6720 was broken down by chelating off calcium.^[^ ^100^, ^103^ ^]^ The binding of calcium with cardiolipin, a major lipid component in Gram‐positive bacteria *Staphylococcus aureus* (*S. aureus*) membranes, was also observed.^[^ ^104^ ^]^ The cell membrane activity of *S. aureus* was disrupted when excessive stimulation with calcium (40 mm calcium chloride).^[^ ^105^ ^]^ Therefore, the conclusion can be smoothly drawn that calcium plays a dual role in bacterial membrane structure, respectively serving as a structural former and potential mediator for maintaining and disrupting the integrity of the bacterial membrane.

**FIGURE 4:**
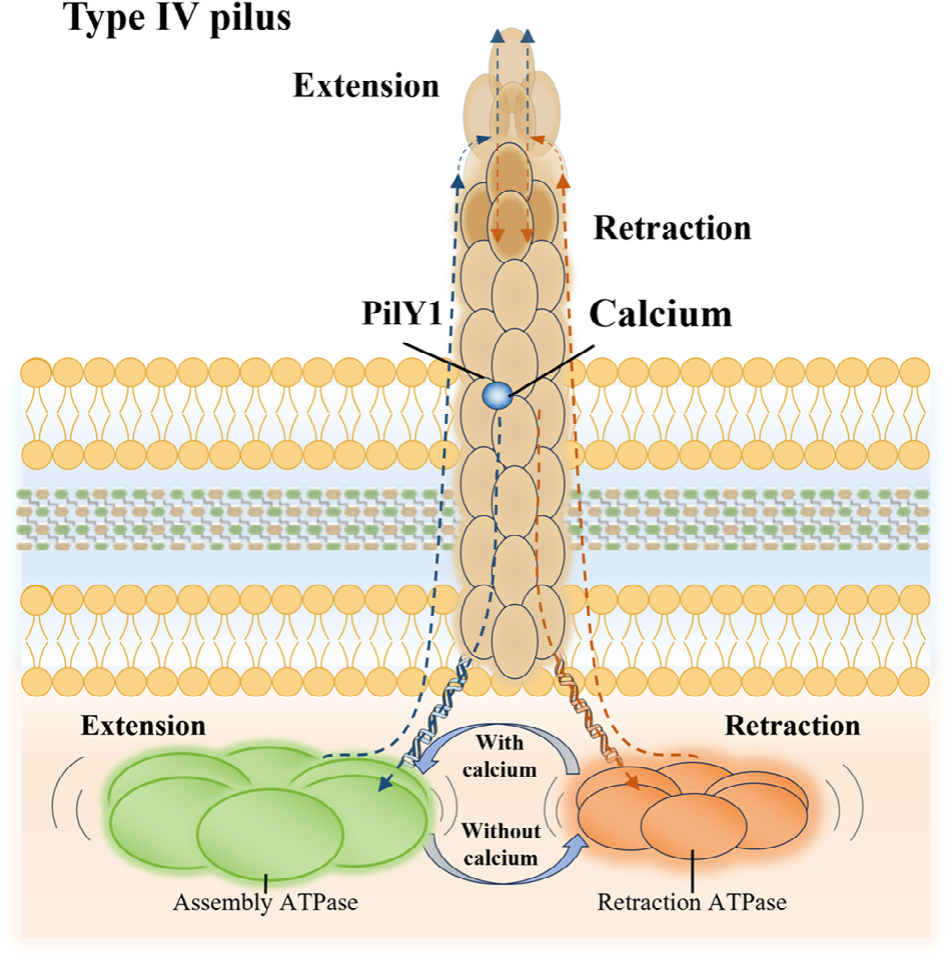
Calcium‐based extensions and retractions of type IV pilus answering for bacterial moves. Calcium serves as a structural trigger for the extension of the pilus‐biogenesis factor (PilY1). Adapted with permission.^[^ ^108^ ^]^ Copyright 2011, MDPI.

Bacteria's mobility is considered critical for their adhesion and dissemination. It was known that bacterial colonization is highly associated with how much calcium is available, just like an optimal calcium concentration for adhesion of *Rhizobium leguminosaram* was found ranging from 3.5 nm to 3.5 mm.^[^ ^106^ ^]^ in vitro studies by Luisa et al. demonstrated that calcium enhanced the motility of *Xylella fastidiosa* (non‐flagellated Gram‐negative). This is because calcium binding contributes to the extensions and retractions of type IV pilus on the bacterial surfaces that facilitate their twitch moving.^[^ ^107^ ^]^ *P. aeruginosa* (a human pathogen) extends and retracts its Type IV pilus to move for infections (Figure 4).^[^ ^108^ ^]^ The pilus‐biogenesis factor (PilY1) in Type IV pilus is a calcium‐dependent regulatory protein, which has a modified β‐propeller fold and an EF‐hand‐like calcium‐binding site for binding (pilus retraction) and dissociation (pilus extension) of calcium.^[^ ^109^ ^]^ Moreover, when the bacteria move to the appropriate location, bacterial flagella can help floating cells overcome electrostatic repulsive barriers for irreversible adhesions,^[^ ^110^ ^]^ and calcium can further bridge interactions between a substrate and those high‐affinity sites on the bacterial surface.^[^ ^111^ ^]^ In the presence of calcium, the bacterial adhesion force has been significantly increased from 160 pN (calcium‐free) to 223 pN.^[^ ^107^ ^]^

**FIGURE 5:**
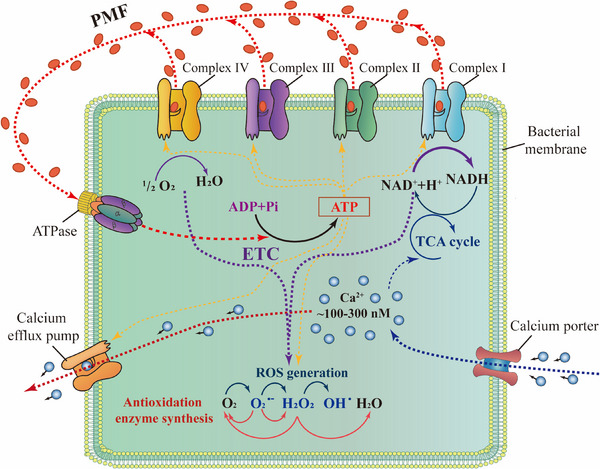
Calcium‐dependent metabolism homeostasis in bacteria. calcium influx stimulates the tricarboxylic acid (TCA) cycle and promotes the overall metabolism in the electron transport chain (ETC), which increases oxygen consumption and accelerates reactive oxygen species (ROS) generation. Proton motive force (PMF); Nicotinamide adenine dinucleotide hydrate (NADH); Adenosine triphosphate (ATP); Adenosine diphosphate (ADP). Adapted with permission.^[^ ^70^ ^]^ Copyright 2018, The Royal Society of Chemistry.

Bacterial metabolism is maintained by the respiration chain in their plasma membrane. The respiration chain is made up of a series of protein complexes, that is, complex I, II, III, and IV (Figure 5),^[^ ^70^ ^]^ which establish a transmembrane proton motive force (PMF) driving the ATP synthase (ATPase) to produce adenosine triphosphate (ATP),^[^ ^112^, ^113^ ^]^ while generates (initiated during the production of nicotinamide adenine dinucleotide hydrate, NADH) an flow of electrons leading to the generation of reactive oxygen species (ROS).^[^ ^94^, ^114^ ^]^ An appropriate ROS level is essential for maintaining the bacteria's signaling systems, whereas disruption of the ROS homeostasis poses detrimental effects on bacterial growth, and even leads to cell deaths. It was found that the ROS generation in bacteria was apparently accelerated by using high extracellular calcium (5 mм of calcium chloride),^[^ ^115^ ^]^ which rapidly increased the intracellular calcium (the normal calcium concentration in a rest bacterium is approximately 100–300 nм) and further pushed the NADH production via the calcium‐involved tricarboxylic acid (TCA) cycle.^[^ ^115^, ^116^ ^]^ Although the intracellular calcium level is tightly regulated by various bacterial calcium pumps via consuming ATP,^[^ ^117^, ^118^ ^]^ high extracellular calcium (10–25 µм) still accelerated the incorporation of phage DNA into *Escherichia coli* strains (*E. coli* K12 and *E. coli* C), indicating high calcium levels likely induce lysis in *E. coli* cells.^[^ ^119^ ^]^ The synthesis of cyclic dinucleotides such as bis(3′−5′)‐cyclic dimeric GMP (c‐di‐GMP) and Vibrio positive regulator (VpsR) in *Vibrio cholerae* was found to actively increase over the presence of 10 mм calcium, demonstrating that the mobility and biofilm formation capability of the microbes were impaired.^[^ ^120^, ^121^ ^]^ This evidence laid promising calcium‐based pathways against bacterial colonization for implantable medical devices.

### 4.2 Calcium in virus

**FIGURE 6:**
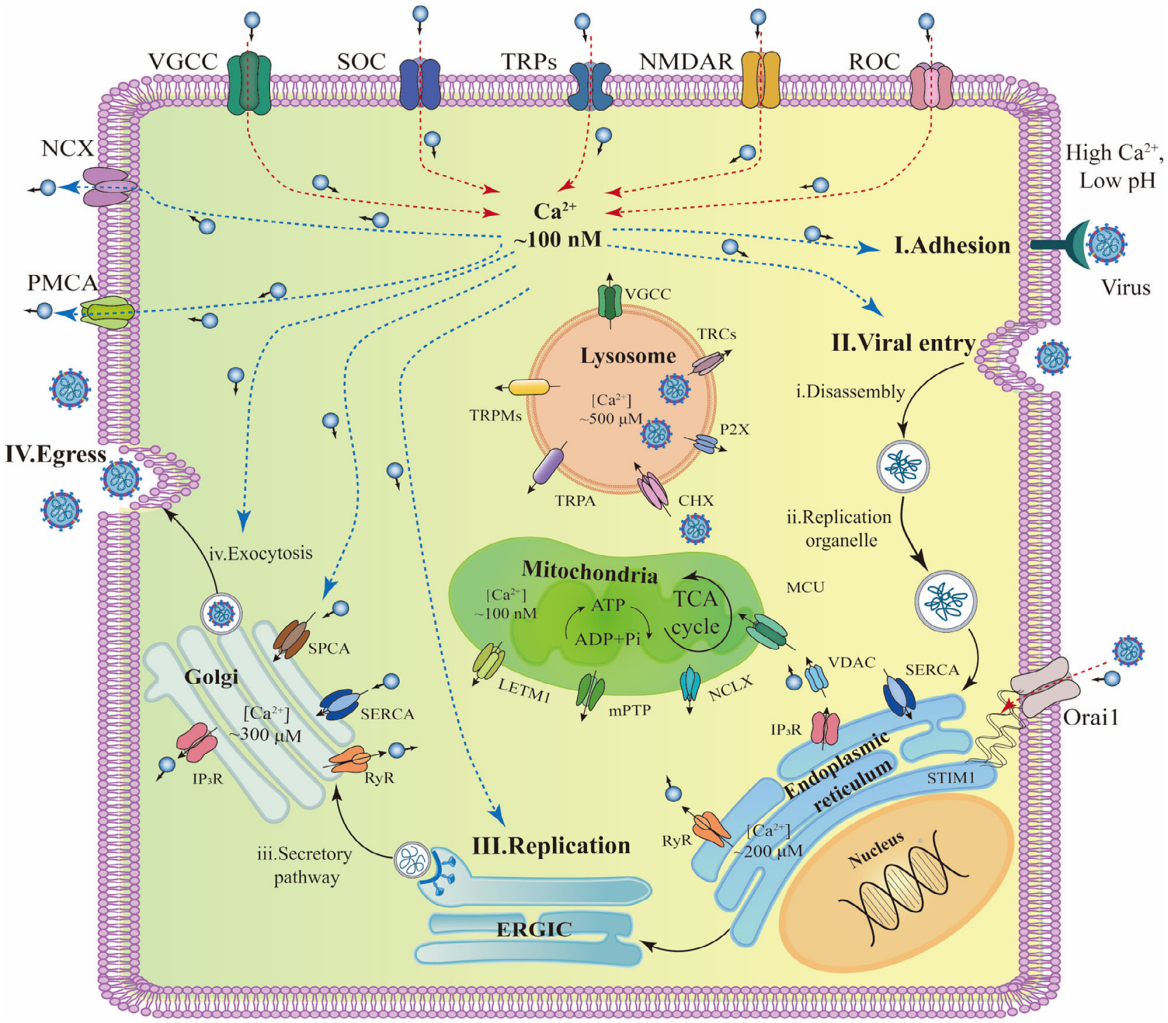
Calcium is involved in the four crucial stages of a viral infection: (I) adhesion, (II) entry, (III) replication, and (IV) egress. Voltage‐gated calcium channels (VGCCs); Store‐operated calcium entry (SOC); Transient receptor potential (TRPs); *N*‐methyl‐d‐aspartate receptors (NMDAs); Receptor operated channel (ROC); Sodium /calcium exchanger (NCX); Plasma membrane calcium pump (PMCA); Mitochondrial calcium uniporter (MCU); Sodium/calcium/ lithium exchanger (NCLX); Secretory pathway calcium ATPase (SPCA); Ryanodine receptors (RyR); Sarcoplasmic/endoplasmic reticulum calcium ATPase (SERCA); Calcium/hydrogen exchangers (CHX); Two‐pore channel 2 (TPC2); Endoplasmic reticulum‐Golgi intermediate compartment (ERGIC); Voltage‐dependent anion channels (VDAC). Adapted with permission.^[^ ^122^ ^]^ Copyright 2020, MDPI.

The virus is an organic structure consisting of nucleic acids and protein coats but lacking intact cellular organization. Consequently, viruses must exploit multiple systems within host cells for energy and metabolism assistance.^[^ ^20^, ^122^ ^]^ Calcium is crucial to all four key stages of viral infection (Figure 6),^[^ ^122^ ^]^ that is, adhesion, entry, replication, and egress; therefore calcium pumps or storage organelles are frequent hijacking targets for viruses.^[^ ^123^ ^]^ Virus appears to induce a receptive state in host cells by assuming an “*acclaimed*” role, thereby facilitating a strong interaction (adhesion) between host cell membrane surface receptors and packaged viruses through the elevation of intracellular calcium levels and subsequent activation of downstream protein calmodulin (CaM).^[^ ^124^ ^]^ The virus tends to transform into a stable conformation following fusion, protecting its ability to withstand the repulsion encountered from both the virus and cellular membranes during intracellular entry, particularly in a conducive environment with the presence of calcium.^[^ ^125^ ^]^ The replication and assembly of the virus are subsequently considered the most crucial steps in its life cycle. Not only does the virus need to exploit multiple organelles within host cells, but it also relies on calcium channels and signaling pathways to acquire calcium.^[^ ^126^, ^127^ ^]^ This regulation involves concomitant control over the permeability of voltage‐dependent anion channels (VDAC) and calcium‐dependent synthesis of reactive oxygen species (ROS), aiming to prevent cell‐induced apoptosis during the early stages of viral infection.^[^ ^128^, ^129^ ^]^ In addition to hijacking the mitochondria, the endoplasmic reticulum (ER), Golgi apparatus, and their intermediate component (ERGIC, the endoplasmic reticulum‐Golgi intermediate compartment) serve as the primary sites for viral replication, assembly, and maturation. The ER is believed to be the site of origin where numerous viral replication complexes have been detected within organelles, shielding them from cellular immune molecules.^[^ ^130^ ^]^ The enhanced fusion of lysosomes and the plasma membrane is the last step to promote viral egress, which is also mediated by elevating the cytosolic calcium levels.^[^ ^131^ ^]^

Take the Coronavirus Disease 2019 (COVID‐19) as an example. The coronavirus consists of four key structural proteins, that is, membrane (M), nucleocapsid (N), envelope (E), and spike (S) proteins, which are responsible differently for its infection.^[^ ^132^, ^133^, ^134^ ^]^ To enter a host cell, the S1 subunit in the S protein interacts with the angiotensin I converting enzyme 2 (ACE2) on the cell membrane, providing a therapeutic target for the treatment of COVID‐19 infection. The shed form of ACE2 is believed a factor determining susceptibility to COVID‐19 pathologies; however, ACE2 shedding is associated with calmodulin (CaM) regulated by calcium levels.^[^ ^135^, ^136^ ^]^ A recent study showed that the S protein changes its conformation favoring virus‐host fusion at high calcium concentrations ranging from 100 to 500 µм (pH = 4.6).^[^ ^125^ ^]^ After cell entry, the virus promptly triggers the decomposition program to facilitate the replication of genomic RNA and structural proteins, which are mainly transported to the ER‐Golgi intermediate compartment (ERGIC) for virus assembly.^[^ ^137^ ^]^ During this process, a significant portion of E protein is involved in the assembly and budding of the virus within ERGIC,^[^ ^138^, ^139^ ^]^ and its power can be enhanced by a calcium stimulus (30 mм of calcium chloride, pH 4.5).^[^ ^140^ ^]^ Additionally, the open reading frame 3a porin (ORF3a) in the COVID‐19 virus normally targets the host cell's ion channels to induce abnormal permeation.^[^ ^141^ ^]^ The expression of ORF3a increases as the calcium concentration is elevated, which enhances the fusion of lysosomes and the plasma membrane, promoting lysosomal exocytosis and viral egress.^[^ ^142^ ^]^ In addition, calcium‐dependent molecules, such as membrane glycoprotein gp120 and transcriptional trans‐activator Tat, are found on the surface of the human immunodeficiency virus (HIV).^[^ ^143^ ^]^ These molecules can trigger the activation of l‐type voltage‐sensitive calcium channels (VGCCs) on the host cell membrane for calcium efflux,^[^ ^144^ ^]^ facilitating the production of pro‐inflammatory cytokine tumor necrosis factor‐alpha (TNF‐α), activation of nuclear factor‐kappa B (NF‐κB), and replication of HIV‐1.^[^ ^145^, ^146^ ^]^ As a result, calcium level control becomes a comment method to treat virus infections. Calcium channel blockers, such as tetrandrine were produced to block the two‐pore calcium channel 2 (TPC2) on lysosomal surfaces and inhibit the entry of COVID‐19;^[^ ^147^ ^]^ nifedipine was developed to block the VGCCs and alleviate the HIV‐induced infection.^[^ ^88^ ^]^

### 4.3 Calcium in fungi

**FIGURE 7:**
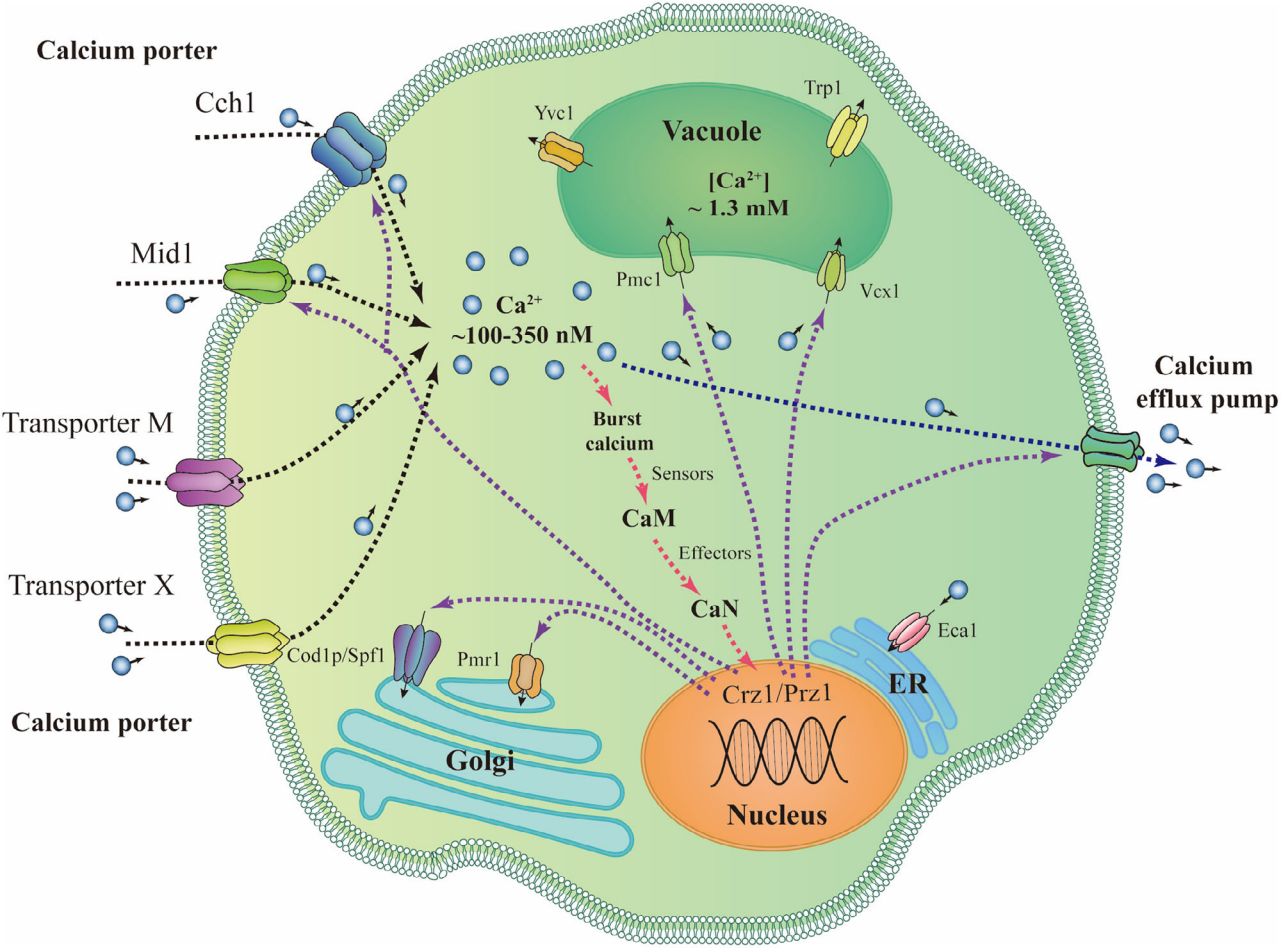
Calcium's circulation and signaling pathways in fungal cells. Yeast vacuole calcium conductance ion transporter (Yvcl); Transient receptor potential channels 1 (Trp1); Vacuolar calcium/ hydrogen exchanger (Vcx1); Vacuolar calcium ATPase pump (Pmc1); Calmodulin (CaM); Calcineurin (CaN). Adapted with permission.^[^ ^151^ ^]^ Copyright 2022, MDPI.

Fungal cells, being eukaryotic, conserve biochemical, and molecular networks shared by all eukaryotes that challenge the treatment of fungal infections.^[^ ^148^ ^]^ The cytosolic calcium in fungi is low, ranging from 100 to 350 nм,^[^ ^149^, ^150^ ^]^ which is controlled by a series of influx and efflux calcium pumps or calcium reservoirs (Figure 7).^[^ ^151^ ^]^ The primary organelle vacuole, rather than ER in mammalian or bacterial cells, stores approximately 90% of the total cellular calcium (∼1.3 mм),^[^ ^150^ ^]^ serving as reservoirs for calcium regulation. There are two types of calcium influx systems, the Cch1 and Mid1 channels are high‐affinity pathways for calcium entry.^[^ ^152^ ^]^ Low‐affinity channels, transporter X and transporter M are also present on the fungal membrane in response to extracellular high calcium.^[^ ^153^ ^]^ In response to high cytoplasmic calcium levels, calcium transport from cytoplasm to vacuoles occurs by using a vacuolar calcium ATPase pump (Pmc1) and vacuolar calcium/ hydrogen exchanger (Vcx1),^[^ ^154^ ^]^ or by activating the efflux calcium pumps in the fungal membrane. In response to high cytoplasmic calcium levels, reversed calcium transport in the vacuole occurs by activating yeast vacuole calcium conductance ion transporter (Yvcl) and transient receptor potential channels 1 (Trp1).^[^ ^155^ ^]^ In addition, calcium transporters, such as Eca1, Cod1p/Spf1p, and Pmr1 in the ER membrane or Golgi apparatus are also applied to assist calcium regulation.^[^ ^156^ ^]^ A study demonstrated that the intracellular calcium level increased approximately 6–10 folds upon the extracellular stimulation by 100 mм calcium chloride, leading to the inhibition of the aforementioned influx of calcium channels Cch1 and Mid1 calcium through dephosphorylation.^[^ ^157^, ^158^ ^]^ The resistance of *Candida albicans* to azoles (anti‐fungal medicine) was attributed to the activation of these two channels by fungi.^[^ ^159^ ^]^ The antifungal efficacy of azoles is dependent on the cytoplasmic calcium levels, and a relatively high calcium concentration normally lowers the pharmacological effect of this drug. To eliminate the resistance, calcium inhibitors, such as ethylene glycol tetraacetic acid (EGTA), fluphenazine, and cyclosporin are usually used to inhibit calcium influx by fungi.^[^ ^160^ ^]^ A clinical study demonstrated that the use of azole itraconazole enriches the intracellular calcium concentration in fungi by transient activation of their Cch1/Mid1, Pmr1, and Pmc1, and blocking these calcium influx channels remarkably increases the antifungal efficacy of the agent.^[^ ^161^ ^]^ Moreover, ambroxol hydrochloride (ABH) can act against multiple pathogens, including viruses, bacteria, and fungi. A recent study demonstrated that the administration of ABH activates multiple calcium channels (Cchl and Yvc1) in the microbes and elevates their intracellular calcium, which undermines the ABH's efficacy.^[^ ^162^ ^]^ These findings demonstrate that calcium homeostasis reconfiguration is a leading pathway for fungi in response to the challenge of traditional antifungal agents.

## 5 CALCIUM'S USE BY THE IMMUNE SYSTEM

Calcium plays a crucial role in mediating the innate immune‐killing and wound‐healing responses in humans. This includes facilitating immune cell migration to injured sites, activating various immune systems, and promoting wound healing. Calcium is involved in nearly all essential immune regulatory processes through distinct pathways based on its unique biochemical properties. These encompass several representative cells and proteins such as neutrophils, lymphocytes, platelets, antimicrobial peptides, calprotectin, and fibrin(ogen).

### 5.1 Calcium's uses in immune cell

**FIGURE 8:**
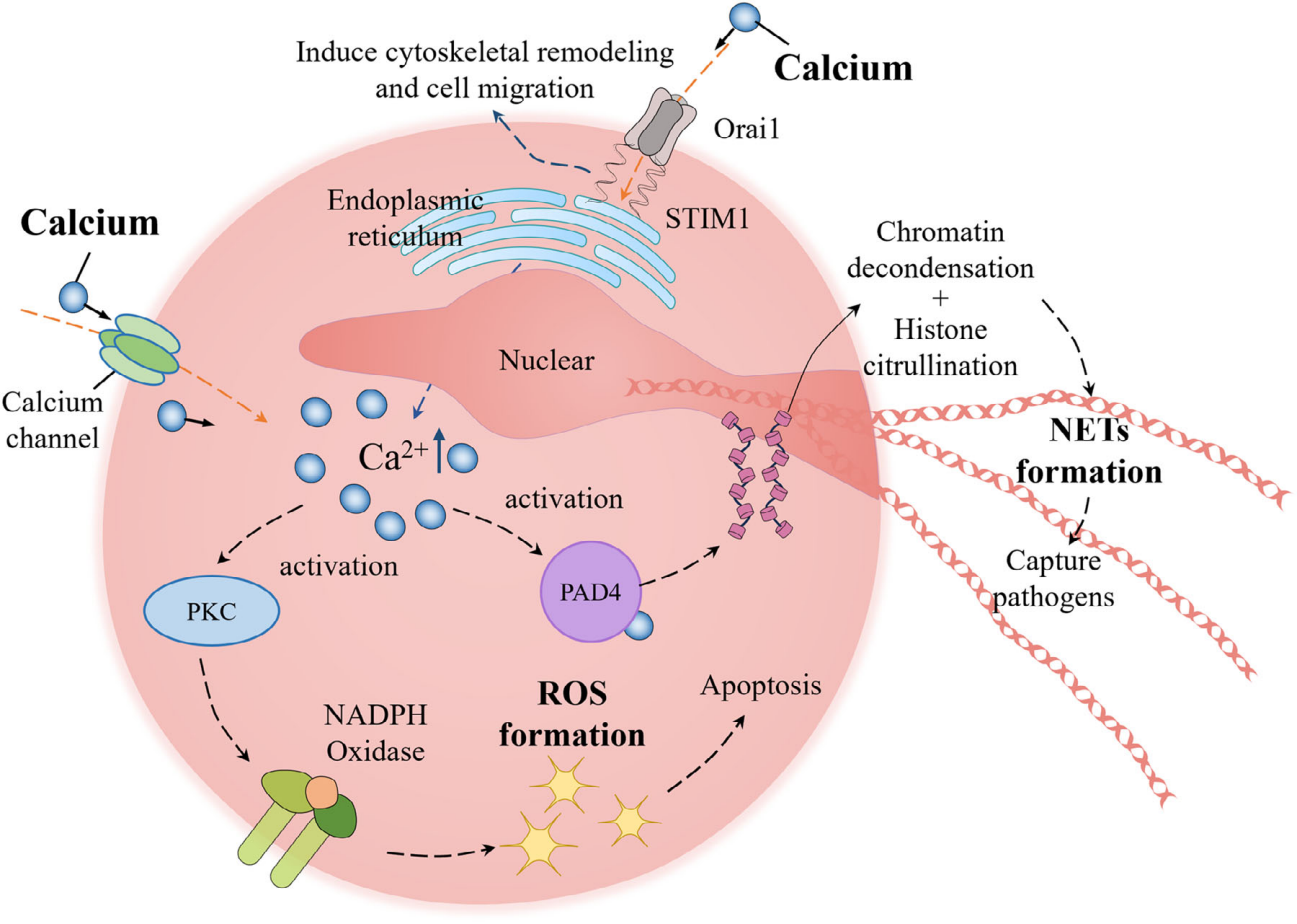
The structural trigger roles of calcium in neutrophils: The influx of calcium via the calcium release‐activated calcium modulator 1 (Cracm1, also known as Orai1) channel mediates neutrophil migration; The elevated cytoplasmic calcium activates the protein‐arginine deiminase type 4 (PAD4) and protein kinase C (PKC), facilitating the generation of neutrophils extracellular traps (NETs) and reactive oxygen species (ROS). Stromal interaction molecule 1 (STIM1). Adapted with permission.^[^ ^163^ ^]^ Copyright 2021, MDPI.

Immune cells are essential components of the human immune system, playing a pivotal role in the recognition and eradication of foreign invaders. The neutrophils and macrophages serve as the body's primary defense mechanism in the immune response. These cells transition from regular patrolling to coordinated recruitment upon tissue damage, followed by a series of overlapping processes including cellular infiltration, secretion of antibacterial proteins, phagocytosis, and clearance. Calcium involves all these processes (Figure 8).^[^ ^163^ ^]^ Calcium serves as a structural trigger for multiple immunoregulatory processes in neutrophils. Migration of neutrophil cells towards the infection or injury sites indicates the initiation of inflammatory reaction, playing a crucial role in facilitating tissue repair and preventing infection. The activation of calcium release‐activated calcium modulator 1 (Orai1) facilitates local cytoskeletal remodeling and enables immune cell migration to specific destinations.^[^ ^164^ ^]^ Calcium uptakes via channels STIM1/Orai1 regulate the release of neutrophil superoxide via activation of protein kinase C isoforms a and b (PKCa and PKCb) and phosphorylation of nicotinamide adenine dinucleotide phosphate (NADPH) oxidase.^[^ ^165^ ^]^ Reactive oxygen species (ROS) generation by NADPH oxidase is a primary bactericidal mechanism. Calcium can activate and degranulate NADPH oxidase, generating antimicrobial ROS.^[^ ^166^ ^]^ The formation of extracellular traps composed of intertwined double‐stranded DNA, antimicrobial peptides, histones, and proteases is a sophisticated pathway in neutrophils for the capture of pathogens and dissemination prevention.^[^ ^24^, ^167^ ^]^ Extracellular trap formation via chromatin decondensation and histone citrullination driven by protein–arginine deiminase type 4 (PAD4), which is motivated by elevated cytoplasmic calcium.^[^ ^168^, ^169^, ^170^, ^171^ ^]^ It was reported that calcium signaling via connexin‐43 (Cx43) hemichannels mediates active ATP release and accelerates chemoattractant synthesis, enhancing the *P. aeruginosa* clearance efficiency by neutrophils.^[^ ^172^ ^]^ In addition, calcium is necessary for phagolysosome fusion and oxidative activation in neutrophils and macrophages.^[^ ^173^, ^174^, ^175^, ^176^ ^]^ For example, the *S. aureus* elimination efficiency by neutrophils can be enhanced by increasing the cytoplasmic calcium to facilitate the activation of killing action proteins.^[^ ^177^ ^]^ Calcium also regulates T and B lymphocyte (T‐ and B‐cells) activation and associated effector functions. T‐ and B‐cells have similar calcium channels, which control their intricate immune responses via multiple signaling interactions.^[^ ^178^ ^]^ The resting cytoplasmic calcium in lymphocyte cells is approximately 100 nм, while this concentration can be increased over tenfold by the engagement of their immunoreceptors.^[^ ^178^ ^]^ Sequential activation of inositol‐1,4,5‐trisphosphate (InsP3) in the endoplasmic reticulum (ER) and calcium channels in the plasma membrane (store‐operated calcium channels, SOC; calcium‐release‐activated calcium channels, CRAC) serves as the primary pathway to elevate the calcium flow into the intracellular space.^[^ ^179^ ^]^ It was reported that short‐term calcium signaling is necessary for granule exocytosis and target killing.^[^ ^180^ ^]^ The interaction between T cells and antigen‐presenting cells bearing antigenic peptides induces a rapid increase of cytoplasmic calcium. This restrains T cells migration facilitating stable immunological synapse formation and ensuring their long‐term functionality.^[^ ^181^ ^]^ These studies indicate that calcium is an effective trigger or regulator exerting immune cells’ gene expression and subsequent cell functions.

Platelets, the smallest and most numerous circulating immune cells in the blood, are derived from megakaryocytes in the bone marrow.^[^ ^182^ ^]^ Platelets act as quiescent soldiers in the peripheral bloodstream circulation, always prepared to respond to organizational injuries and microbial threats. They are also responsive cells bridging hemostasis in an injured area and the host immune defense.^[^ ^183^ ^]^ Notably, calcium is also a regulator in platelet migration, activation, and phagocytosis.^[^ ^184^ ^]^ Platelets equipped with a variety of sensors and pattern recognition receptors enable rapid detection of tissue injuries and microbial threats.^[^ ^183^ ^]^ Therefore, platelets largely and swiftly migrate to and aggregate at trauma and infection sites.^[^ ^185^ ^]^ The migratory response of platelets, similar to that of neutrophils, is linked to the activation of the calcium‐induced channel Orai1 and the calcium‐sensitive potassium ion channel.^[^ ^186^ ^]^ Platelets have open‐canalicular or surface‐connected systems (SCS) and dense tubular systems (DTS), which provide sophisticated networks for the transmission of internal and external substances.^[^ ^29^ ^]^ The DTS system contains calcium‐ATPase and intracellular calcium channel IP3, which controls the influx of extracellular calcium for efficient calcium homeostasis regulation.^[^ ^187^, ^188^ ^]^ Platelets possess a strong calcium affinity and calcium demand for commencing immunomodulatory functions.^[^ ^189^, ^190^ ^]^ Calcium plays a pivotal role in platelet activation and host‐defense peptide releases. These peptides, such as platelet microbicidal proteins, defensins, and kinocidins, exhibit rapid and potent effects against viral, bacterial, and fungal infections.^[^ ^183^, ^191^ ^]^ This process can be divided into three distinct phases. The first phase is calcium‐induced rapid shape change in platelets,^[^ ^192^ ^]^ which alters the cytoskeleton facilitating function‐responsive activation, like new actin filaments formation.^[^ ^193^ ^]^ Then elevating the cytoplasmic calcium level via the DTS systems in platelets,^[^ ^194^ ^]^ which stimulates secretory exocytosis promoting platelets interaction.^[^ ^195^ ^]^ The final phase is a rearrangement of the platelet membrane to increase calcium permeability, which enables a continuous supply of calcium for tissue healing.^[^ ^196^ ^]^ During activation, platelets undergo a structural transformation from spheroid to amoeboid with pseudopodia, enabling internalization and elimination of pathogens.^[^ ^197^ ^]^

### 5.2 Calcium's uses in immune proteins

Antimicrobial peptides (AMPs) are short polypeptides consisting of fewer than 100 amino acids, naturally occurring in host defense and exhibiting antimicrobial activity at physiological concentrations within the host tissues.^[^ ^198^ ^]^ Defensins are a class of small cationic antimicrobial peptides, typically consisting of 29–45 amino acid residues and having molecular weights ranging from approximately 3 to 6 kDa.^[^ ^199^ ^]^ They extensively distribute in mammalian epithelial cells and phagocytes, with their concentrations reaching millimolar levels when it is necessary.^[^ ^200^ ^]^ The α‐defensins released by human polymorphonuclear neutrophils (e.g. human neutrophil peptides, HNP) are closely related to an immediate calcium influx mediated by pathogen binding.^[^ ^201^ ^]^ The β‐defensins, another group of members in the innate immune system, act as chemoattractants in mast cells, immature dendritic cells, and T‐lymphocytes, which establish a connection between the innate and acquired immune responses.^[^ ^202^ ^]^ Four human β‐defensin (hBD1, hBD2, hBD3, and hBD4) have been identified in epithelial tissue. The hBD1 is widely distributed in various epithelial cells and tissues, including those of the oral cavity.^[^ ^25^ ^]^ The hBD2, initially discovered in psoriatic skin, is also expressed in oral epithelia.^[^ ^203^ ^]^ Both hBD1 and hBD2 exhibit a broad spectrum of antimicrobials both Gram‐negative and Gram‐positive bacteria in vitro.^[^ ^204^ ^]^ The hBD3 detected in oral tissues shows greater specificity towards Gram‐positive organisms.^[^ ^205^ ^]^ Importantly, the production of peptide hBD2 in oral epithelial cells can be enhanced with high calcium levels ranging from 0.60 to 1.20 mм.^[^ ^206^ ^]^ The sufficient expression of antimicrobial peptide hBD3 can be triggered by elevating the calcium concentrations, particularly at a concentration of 1.7 mм.^[^ ^207^ ^]^ In addition, the expression of interleukin‐8 (IL‐8, an inflammatory cytokine) is involved in neutrophils’ morphology changes, chemotaxis, granule release, and activation.^[^ ^208^ ^]^ A study demonstrated that the expression of IL‐8 was upregulated in a dose‐dependent manner as the extracellular calcium concentrations increased.^[^ ^206^ ^]^

**FIGURE 9:**
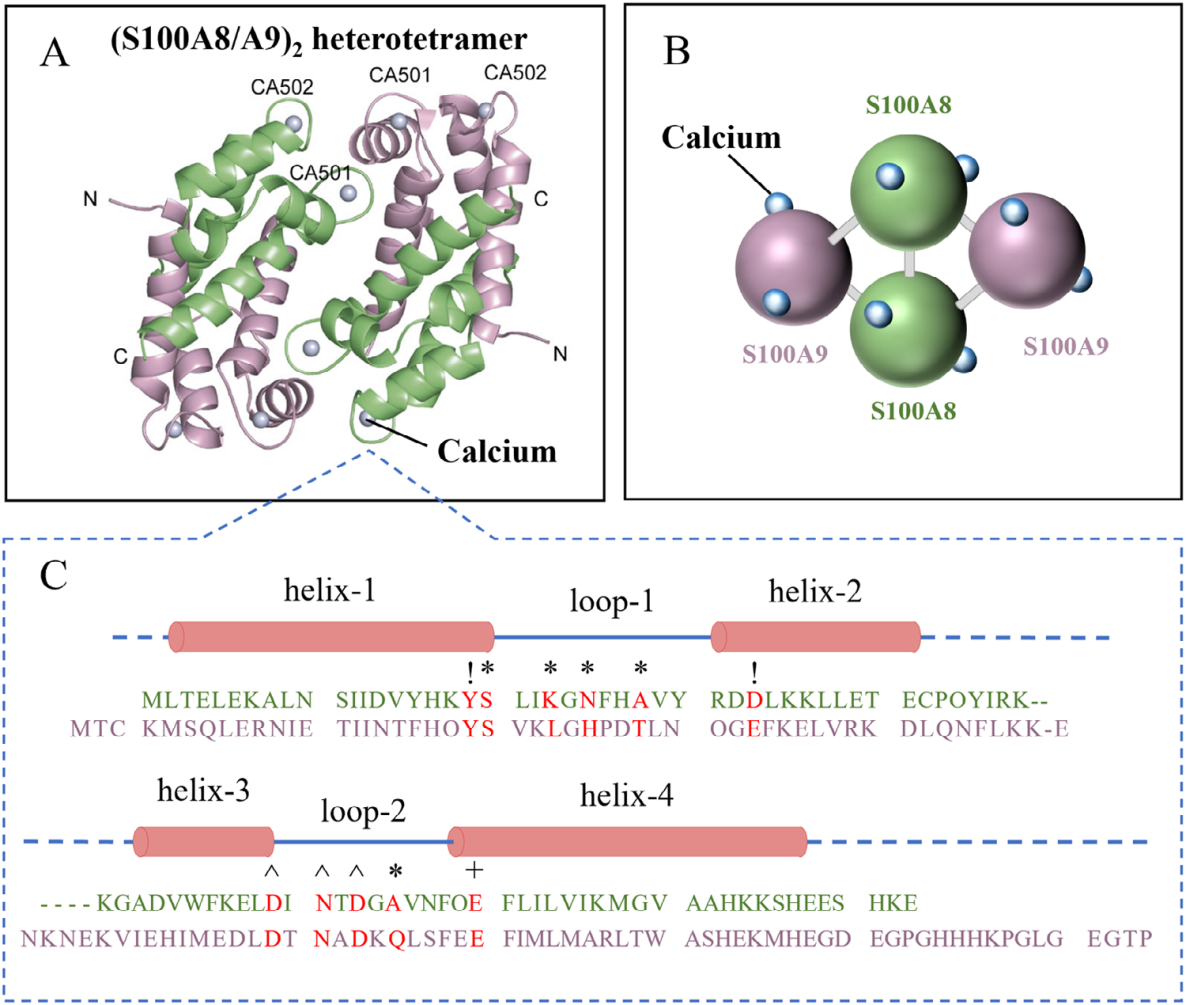
Calcium acts as a structural former in calprotectin S100A8/A9 heterotetramer: (A) The calcium‐induced (S100A8/A9)_2_ heterotetramer calprotectin formation. S100A8, green; S100A9, pink; Calcium, light blue spheres. Reproduced with permission^[^ ^220^ ^]^. Copyright 2007, Elsevier. (B) Schematic diagram of the arrangements of calcium and S100SA8 and S100A9 in calprotectin. (C) The calcium‐binding loops on amino‐acid sequences of S100A8 and S100A9. The residues (red color) coordinate calcium are indicated as follows: *, backbone carbonyl group; !, water‐mediated; ^, monodentate side chain of Asp or Asn; +, bidentate side chain of Glu. Reproduced with permission.^[^ ^221^ ^]^ Copyright 2004, Blackwell Munksgaard.

The antimicrobial protein calprotectin (CP), as a congenital defense mechanism directly targeting pathogens, is abundantly present in immune cells such as neutrophils and macrophages. Upon pathogens invading, this protein is rapidly released by immune cells. It has become a reliable biomarker for the acute activation of inflammatory cells and the diagnosis of joint infections.^[^ ^209^ ^]^ The protein also serves as a potential immunomodulatory molecule involved in the activation of toll‐like receptor 4 (TLR4) and engagement of CD69.^[^ ^210^, ^211^ ^]^ Protein CP belongs to the S100 family, which consists of approximately 25 subsidiary subunits, each of them possessing two calcium‐binding EF‐hand motifs.^[^ ^212^ ^]^ Five calprotectin members in the S100 family, that is, S100A7 (Psoriasin), S100A8, S100A9, S100A12, and S100A15 are involved in antibacterial defense. These proteins can confine bacterial growth by sequestering metals available at the site of infection.^[^ ^213^, ^214^, ^215^, ^216^ ^]^ The amino acids 35–80 within the central region of S100A7 exhibit significant antibacterial activity by reducing bacterial adhesion at infection sites.^[^ ^217^ ^]^ Elevated intracellular calcium levels induce a conformational change in the homodimer of S100A7, exposing the hydrophobic binding domain and creating a more stable structure for antimicrobial defense.^[^ ^218^ ^]^ In the presence of calcium, proteins S100A8 (α subunit) and S100A9 (β subunit) undergo self‐assembly (S100A8 and S100A9 have two binding loops for calcium), forming a heterotetramer (*α_2_β_2_*) other than a heterodimer (*αβ*) (Figure 9).^[^ ^219^, ^220^, ^221^ ^]^ The former structure has antibacterial activity while the latter one does not. This structural change is a result of calcium‐induced heterotetramerization, which protects CP from protease degradation and promotes nutritional immunity.^[^ ^215^, ^222^ ^]^ Calcium and zinc jointly induce structural changes in protein S100A12, producing significant chemical shift perturbations to the helix II and the hinge region. These changes confer the protein‐enhanced survival capacity under specific inflammatory conditions and diseases.^[^ ^223^ ^]^ Protein S100A12 exhibits an exceptional ability to sequester zinc at neutral pH conditions. This sequestration scope expands to pH 5.7 when connective calcium brings β‐strand conformation into S100A12's EF‐hand loop, improving the protein's resistance to pH changes.^[^ ^223^, ^224^ ^]^ In fact, calcium‐mediated structural change in EF‐hand domains and associated transition metal sequestration (iron, manganese, zinc, and nickel) is a frequent pathway that accelerates heterotetramers formation and favors the antibacterial capacity of the S100 family calprotectin.^[^ ^225^, ^226^, ^227^ ^]^ For example, neutrophil‐derived calprotectin inhibited *S. aureus* growth through the chelation of manganese and zinc from the bacterial cells. The antibacterial efficacy of calprotectin can be strengthened by purifying the protein in the presence of calcium.^[^ ^225^, ^226^, ^227^ ^]^ This is because calcium plays a structural former role in calprotectin S100A8/A9 heterotetramer.^[^ ^225^, ^226^, ^227^ ^]^

The protein fibrinogen served as a coagulation factor in human blood plasma, typically present at concentrations of approximately 1.5–4 g L^−1^. Both fibrinogen and its derivative fibrin have overlapping functions in various processes including blood clotting, fibrinolysis, inflammation, and wound healing.^[^ ^228^ ^]^ The fibrinogen molecule consists of three distinct polypeptide chains, namely Aα, Ββ, and γ chains. These chains form elongated structures of 45 nm in length, which feature globular domains positioned at both ends (denoted as D domains) and in the middle (denoted as E domains). These domains are interconnected by coiled‐coil segments and linked by disulfide bonds.^[^ ^229^ ^]^ The E domain cleaves the peptides of Aα and Ββ upon binding thrombin, facilitating fibrinogen assembly into fibrin.^[^ ^230^ ^]^ Calcium‐induced fibrinogen assembly was first reported in 1952 when Rosenfeld and Janszky reported that the fibrinogen to fibrin transformation was restrained by calcium‐chelating agent EDTA, and the transformation was completely recovered by calcium supplementation.^[^ ^231^ ^]^ Subsequent investigations on bovine fibrinogen revealed that multiple residues, such as histidine, tyrosine, and carboxyl residues are involved in generating binding sites with high and low affinity to calcium at pH 7.5.^[^ ^30^, ^232^, ^233^ ^]^ A higher concentration of calcium (0.05 м calcium chloride) can turn the carboxy‐terminal regions of the Aα chains in fibrinogen into a more solvent‐exposed position favorable for intermolecular interactions, which likely promotes the release of FpB40 and exposure of the antimicrobial motifs of the protein.^[^ ^234^, ^235^ ^]^ Very recently, our group prepared calcium‐doped titanium (Ti/Ca) by using a duplex physical vapor deposition procedure. The material demonstrated no apparent effect on inhibiting *P. aeruginosa* (ATCC 27853) adhesion and growth, while it had strong activity on preventing bacterial colonization after being conditioned by fibrinogen adsorption. A calcium concentration‐dependent conformational change was detected in the adsorbed fibrinogen, which we believe is the exposure of the antimicrobial peptide Bβ15‐42 in fibrinogen binding to calcium.^[^ ^35^ ^]^

## 6 CALCIUM'S USES IN INFECTION MEDICATIONS

**FIGURE 10:**
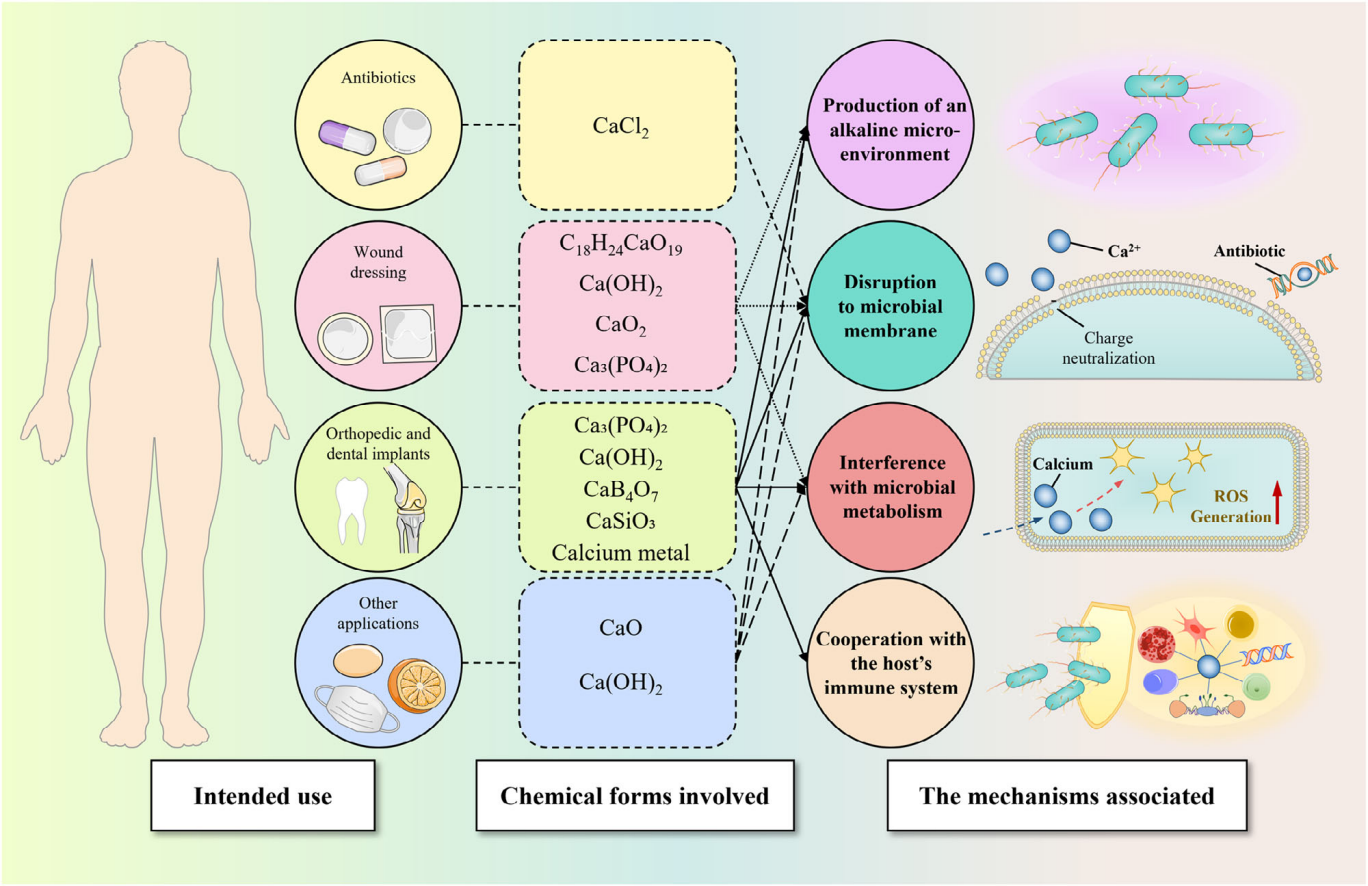
Intended uses of various calcium for disinfection and the mechanisms associated: Calcium hydroxide (Ca(OH)_2_), calcium peroxide (CaO_2_), Calcium oxide (CaO), and calcium metal (change into CaO when exposure to the atmosphere) can produce an alkaline micro‐environment that suppresses microbial growth; Calcium in forms of calcium chloride (CaCl_2_), calcium alginate (C_18_H_24_CaO_19_), Ca(OH)_2_, CaO_2_, CaO, calcium metal, calcium phosphate (Ca_3_(PO_4_)_2_), calcium borate (CaB_4_O_7_), and calcium silicate (CaSiO_3_) can deliver calcium ions that disrupts the microbial membrane, interfere with microbial metabolism, or cooperate with the host's immune system in acting against microbial colonization.

Currently, the intended uses of calcium for disinfection are extended to a range of fields, including antibiotics, wound dressings, orthopedic and dental implants, personal hygiene, veterinary medication, and food processing. The antimicrobial efficacy of calcium varies with specific compounds (Figure 10). Calcium compounds, such as calcium chloride (CaCl_2_), calcium alginate (C_18_H_24_CaO_19_), calcium hydroxide (Ca(OH)_2_), calcium peroxide (CaO_2_), calcium oxide (CaO), calcium metal, calcium phosphate (Ca_3_(PO_4_)_2_), calcium borate (CaB_4_O_7_), and calcium silicate (CaSiO_3_) can deliver calcium ions that disrupt the microbial membrane, interfere with microbial metabolism, or cooperate with the host's immune system in acting against microbial colonization. In addition to the delivery of calcium ions by the compounds, Ca(OH)_2_, CaO_2_, CaO, and calcium metal (change into CaO when exposed to the atmosphere) can produce an alkaline micro‐environment that suppresses microbial growth.

### 6.1 Adjuvants for antibiotics

Antibiotics are frequently prescriptions for bacterial infections; however, their antibacterial efficacy is being challenged by resistant strains, such as methicillin‐resistant *S. aureus* (MRSA), methicillin‐resistant *Staphylococcus epidermidis*, and multidrug‐resistant *S. epidermidis*.^[^ ^236^ ^]^ It is important to develop new antibiotics with a small resistance tendency or to restore the effectiveness of ‘*old*’ antibiotics against bacterial infections. Table 1 lists typical examples of calcium‐dependent antibiotics. Their antibacterial efficacy largely increased in the presence of calcium chloride. The roles of calcium include acting as a structural former in antibiotics for bacterial membrane perturbations, mediating antibiotic interaction with lipid II and the subsequent bacterial wall deactivation, and mediating antibiotic binding to undecaprenyl phosphate (C_55_‐P) and disruption of cell wall biosynthesis. Daptomycin, consisting of ten amino acids macrolactone and an exocyclic linear tripeptide, is a lipopeptide antibiotic capable of destroying the surface integrity of Gram‐positive bacteria.^[^ ^237^, ^238^, ^239^ ^]^ Calcium plays an important role in the interaction between daptomycin and bacteria by triggering a structural transformation in the drug.^[^ ^69^ ^]^ Specifically, the binding of calcium to daptomycin stabilizes the Asp‐7, Asp‐9, and Gly‐5 domains to generate a more compact structure through nuclear Overhauser effect (NOE) restraints.^[^ ^240^ ^]^ The presence of calcium reinforces the interactions between the *N*‐decanoyl fatty acid chain on the bacterial surface and the Trp‐1, D‐Asn‐2, and Kyn‐13 side chains in daptomycin^[^ ^241^ ^]^ The amphipathicity of daptomycin increases as a result of its binding to calcium, thereby facilitating deeper insertion into the *S. aureus* membrane and inducing significant perturbations in the membrane structure.^[^ ^69^ ^]^ The calcium‐dependent oligomerization occurs on liposomes composed of 2R,2′S‐phosphatidylglycerol isomer, particularly in the head group position.^[^ ^242^ ^]^ The calcium‐strengthened aggregation of oligomers (dimers, trimers, and tetramers) produces holes in the phosphatidylglycerol membrane and cell death.^[^ ^243^ ^]^ Therefore, the minimal inhibitory concentration of daptomycin against MRSA was found calcium concentration‐dependent,^[^ ^69^ ^]^ and without the assistance of calcium, daptomycin is unable to be inserted into the neutral and acidic phospholipid bilayers of the *S. aureus* to lethal effect.^[^ ^69^, ^244^, ^245^ ^]^ A very recent study further demonstrated that daptomycin interacts with bacterial cell walls via forming a complex of phosphatidylglycerol and undecaprenyl‐coupled intermediates lipid II upon the assistance of calcium.^[^ ^246^ ^]^ Since lipid II is involved in cell wall assembly, peptidoglycan biosynthesis, and cell division,^[^ ^247^ ^]^ the assistance of calcium will likely make daptomycin defeat bacterial cells with a small chance of resistance.^[^ ^246^ ^]^ Moreover, antibiotics can also achieve desirable antibacterial effects by disrupting the normal biosynthesis of bacterial cell walls. Malacidins A, another group of antibiotics have calcium‐dependent activity against Gram‐positive pathogens, such as methicillin‐resistant *S. aureus* (with a minimum inhibitory concentration (MIC) of 0.2–0.8 µg mL^−1^) and vancomycin‐resistant *Enterococcus faecium* (*E. faecium*, with a minimum inhibitory concentration of 0.8–2.0 µg mL^−1^). The binding of Malacidins A to cell wall precursor lipid II was enhanced in the presence of calcium, and subsequently deactivation of bacterial wall.^[^ ^248^, ^249^ ^]^ Laspartomycin C and cadasides A also were found to have similar calcium‐dependent activity in disrupting the biosynthesis of undecaprenyl phosphate C_55_‐P (the intermediate of the bacterial cell wall) and the integrity of bacterial cell walls. Importantly, their efficacy against *S. aureus* (ATCC 29213, NRS100, and NRS140), *Staphylococcus simulans* (*S. simulans*, ATCC 22), and *E. faecium* (ATCC 51559) was greatly improved by the proper amount of calcium (Table 1).^[^ ^250^, ^251^, ^252^, ^253^ ^]^ These results demonstrated that taking advantage of calcium is a promising pathway leading to developing antibiotics against resistant pathogens.

**TABLE 1:** Typical examples of calcium‐dependent antibiotics.

Antibiotic	Bacterial strain	Compound	Concentration	Administration	Mechanism	Optimal outcomes	Ref.
Daptomycin	*S. aureus*	Calcium chloride (CaCl_2_)	0.34–5 mм	Both calcium and Daptomycin added to the bacterial suspension	Structural former in daptomycin for bacterial membrane perturbations.	The MIC against *S. aureus* decreased from 64 to 0.625 µg mL^−1^. 5 mм CaCl_2_ administrated.	[69]
Malacidin A	MRSA (USA300; BAA‐42; COL)	CaCl_2_	0.25–25 mм	Bacterial suspension was added to the microtiter plates containing calcium and Malacidin A	Mediates antibiotic interaction with lipid II and the subsequent bacterial wall deactivation.	The MIC against MRSA decreased from 100 to 0.2–0.8 µg mL^−1^. 15 mм CaCl_2_ administrated.	[248]
Laspartomycin C	*S. aureus* (ATCC 29213); *S. simulans* (ATCC 22)	CaCl_2_	1.25–10 mм	Added calcium to bacterial suspension with dissolved peptides in microtiter plates	Mediates antibiotic binding to undecaprenyl phosphate (C_55_‐P) and the subsequent suppression of lipid II formation.	The MIC against *S. aureus* and *S. simulans* decreased from 256 to 2 µg mL^−1^, and from 128 to 1 µg mL^−1^, respectively. 10 mм CaCl_2_ administrated.	[251]
Cadasides A	*S.aureus* (NRS100; NRS140); *E. faecium* (ATCC 51559)	CaCl_2_	6.25–400 mм	Bacterial suspension was added to the microtiter plates containing calcium and Cadasides A	Mediates antibiotic binding to C_55_‐P and the subsequent disruption of cell wall biosynthesis.	The MIC against *S. aureus* and *E. faecium* decreased from 128 to 1 and 4 µg mL^−1^ respectively. 100 mм CaCl_2_ administrated.	[253]

### 6.2 Wound dressings

Wound dressings are normally designed to maintain an optimal moist environment for wound healing and provide a physical barrier against bacterial invasion.^[^ ^254^ ^]^ Studies found that calcium alginate is a procoagulant against bacterial invasion because it contains a large number of hydroxyl and carboxyl groups, and simultaneously releases calcium to reinforce additional antibacterial.^[^ ^255^, ^256^, ^257^ ^]^ In a diabetic rat model, a dressing of calcium alginate was demonstrated capable of promoting collagen type I synthesis and re‐epithelialization and mitigating inflammation.^[^ ^258^ ^]^ A recent study demonstrated that, by doping of 0.25 м calcium chloride, alginate's inhibition zone against *E. coli* (ATCC 25922) and *S. aureus* (ATCC 25923) was increased by 11.2 and 9.5 mm, respectively. This was attributed to calcium's disruption effect on bacterial cell membranes and the ability to enhance reactive oxygen species (ROS) generation.^[^ ^259^ ^]^ Moreover, early studies demonstrated that dressings of calcium hydroxide have good efficacy in eradicating and preventing bacterial growth via releasing high levels of hydroxide and calcium ions.^[^ ^260^, ^261^ ^]^ It was demonstrated that the counts of *E. faecalis* inside infected single‐rooted canals were statistically decreased by applying a calcium hydroxide paste (an intra‐canal dressing) for 7 days.^[^ ^262^ ^]^ It was found that the antibacterial activity of a wound dressing against *E. coli* was increased by adding calcium peroxide (13 wt%), which released the maximum value of oxygen during the first day and gradually approached a constant value for the next 7 days.^[^ ^263^ ^]^ Also, the sustained release of hydrogen peroxide_,_ oxygen, and calcium by calcium peroxide (0.5 wt%) doped the hydrogels was found effective in inhibiting the growth of both *E. coli* and *S. aureus*.^[^ ^264^ ^]^ Very recently, a calcium phosphate nanoflakes‐based hemostatic dressing was developed by Akram et al. They found the dressing was highly compatible with blood cell adhesion and underwent immediate blood clotting within a 15 s exposure, and simultaneously the hemostatic dressing was effectively killing or inhibiting the growth of *E. coli* (100 mg of calcium phosphate nanoflakes) and *S. aureus* (200 mg of calcium phosphate nanoflakes).^[^ ^265^ ^]^ These results demonstrated the excluding role of calcium in promoting cutaneous wound healing while shielding the wound from infection.

### 6.3 Orthopedic and dental implants

**FIGURE 11:**
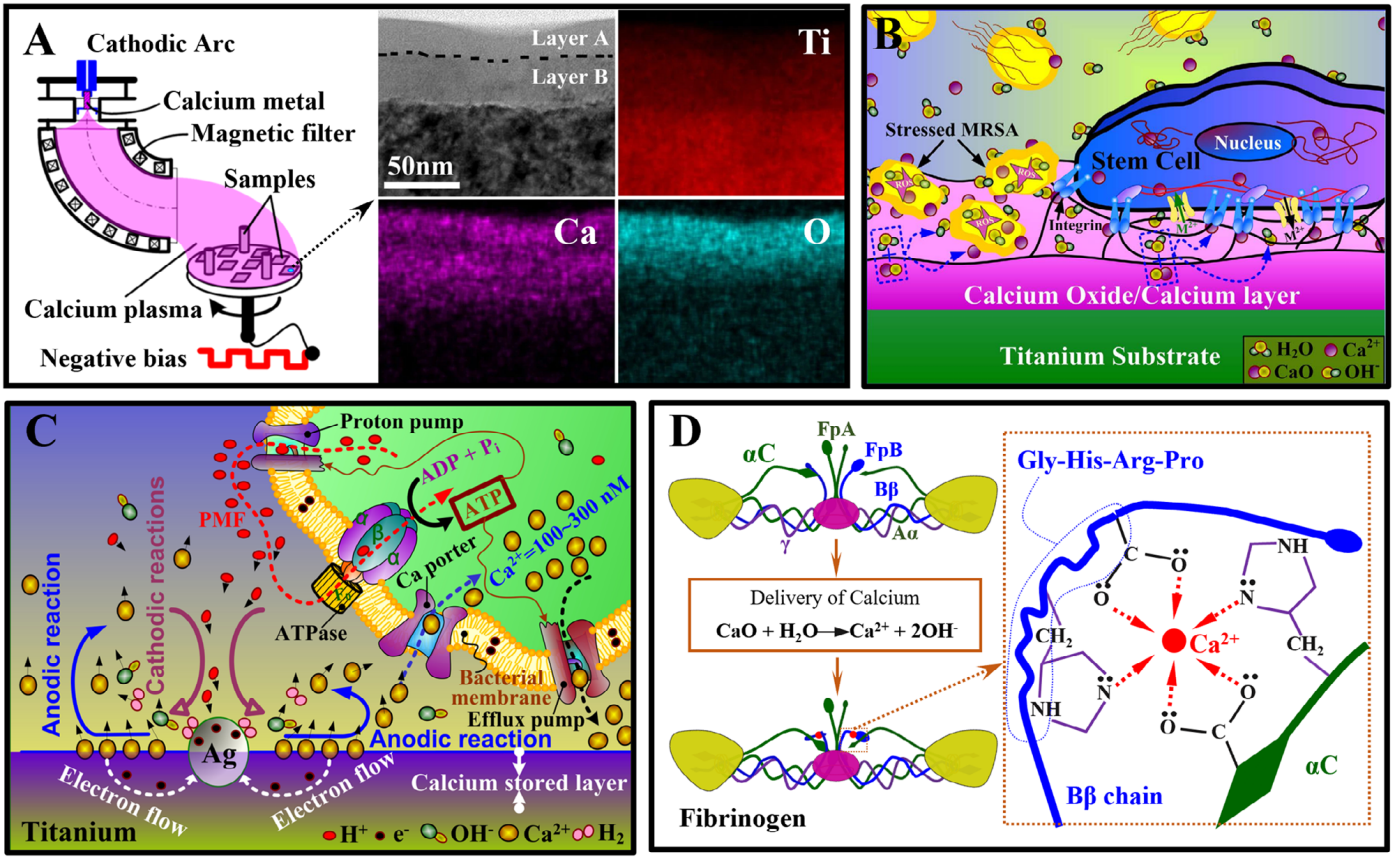
Calcium's uses for disinfection of orthopedic and dental implants: (A) Fabrication of calcium‐doped titanium by using calcium arc sourced plasma immersion ion implantation (Ca‐PIII), together with energy dispersive mapping of the calcium (Ca), oxygen (O), and titanium (Ti) in the samples under cross‐sectional transmission electron microscopy. (B) The calcium‐doped titanium is capable of producing an alkaline micro‐environment and releasing calcium ions that suppress microbial growth. (A and B) are adapted with permission.^[^ ^34^ ^]^ Copyright 2016, Nature Publishing Group. (C) Calcium cooperated with silver (Ag) which can boost the generation of reactive oxygen species in bacteria and disrupt the proton motive force (PMF) on the bacterial surface. Adapted with permission.^[^ ^70^ ^]^ Copyright 2018, The Royal Society of Chemistry. (D) Calcium‐doped titanium can guide the adsorption of fibrinogen and act against bacterial adhesion. Gly–His–Arg–Pro (Gly, glycine; His, histidine; Pro, proline; and Arg, arginine). Adapted with permission.^[^ ^35^ ^]^ Copyright 2022, The Royal Society of Chemistry.

The demand for orthopedic (such as artificial hips and knees) and dental implants is increasing due to the growing elderly population in the world.^[^ ^266^ ^]^ Calcium has proved effective in improving the osseointegration of these medical devices.^[^ ^267^ ^]^ Recently, the antimicrobial activity of calcium was also reported in this field. It not only serves as a physical barrier membrane that spreads over the canal to effectively obstruct bacterial attachment but also causes significant damage by releasing hydroxyl and calcium ions that infiltrate into the bacteria.^[^ ^268^ ^]^ Calcium phosphate serves as a primary constituent of dentin, effectively preventing the infiltration of other substances into the dentinal tubules. Additionally, the released calcium participates in immunoregulation, endowing the material with favorable antibacterial properties.^[^ ^269^, ^270^ ^]^ Calcium phosphate‐based materials also were found capable of regulating the host's inflammatory process and involved in triggering the release of extracellular traps and proinflammatory cytokines in human macrophages for bacterial clearance.^[^ ^271^, ^272^, ^273^ ^]^ Calcium hydroxide is frequently used to eliminate pathogens from the micro‐root canal system. Very recently, Reyhani et al. reported the efficacy of nano‐calcium hydroxide particles for root canal treatment. Due to the small size, the materials were able to penetrate the dentinal tubules, which solved the inaccessible issue of conventional calcium hydroxide with a diameter larger than the dentinal tubules. As a result, the colony‐forming units of *E. faecalis* (ATCC 29212) at week four and week six were reduced approximately 2‐fold and 3.7‐fold, respectively.^[^ ^274^ ^]^ Moreover, calcium borate in a concentration of 0.50 or 0.75 mg mL^−1^ was found effective in preventing bacterial adhesion and inhibiting biofilm formation on orthopedic implants.^[^ ^275^, ^276^ ^]^ The addition of calcium silicate was reported as good for promoting the functions of bone cells while inhibiting bacterial adhesion on the implant surfaces.^[^ ^277^, ^278^ ^]^ Hasna et al. demonstrated that calcium silicate‐based cement was effective against *Porphyromonas gingivalis*, *Porphyromonas endodontalis*, and *Parvimonas micra*, while had good compatibility with macrophages (RAW 264.7) and osteoblasts (MG‐63).^[^ ^279^ ^]^ Although these authors did not elaborate on the antimicrobial mechanism of calcium silicate, we believe this is associated with the material's capability of releasing high calcium concentrations and disrupting the metabolic balance in bacteria. In recent years, our group focused on developing calcium‐doped titanium for orthopedic and dental implants. In one study we introduced metallic calcium into a commercial pure titanium surface by using a cathodic arc‐sourced plasma immersion ion implantation (PIII) process (illustrated in Figure 11A).^[^ ^35^ ^]^ Cross‐sectional transmission electron microscopy observations demonstrated that the doped calcium was well mixed with the titanium substrates (the energy dispersive mapping in Figure 11A). The materials were able to release calcium and generate an alkaline micro‐environment that enhanced bacterial reactive oxygen species (ROS) generation and restored the osteogenic functions in bone marrow stem cells (BMSCs) for osseointegration (illustrated in Figure 11B).^[^ ^34^ ^]^ In another study, we co‐doped titanium of calcium and silver by simultaneously triggering two cathodic arcs during PIII. Galvanic corrosion‐based synergistic effect of calcium and silver (nanoparticles) on inhibiting bacterial growth was evidenced, that is, the anodic reactions on calcium‐stored regions released calcium that elevated the calcium concentration in bacterial cells while the cathodic reactions on silver particles consumed protons that likely disrupted that proton motive force for bacterial energy synthesis (illustrated in Figure 11C).^[^ ^70^ ^]^ More importantly, the released calcium also stimulates immune responses in the host to act against bacterial colonization. Calcium‐doped implants favored the adsorption of platelets and plasma proteins and showed significantly less bacterial adhesion (67% reduction on average) and biofilm formation (40% reduction on average) than the undoped control.^[^ ^32^, ^280^ ^]^ This is consistent with a very recent study of our group, in which we found that calcium‐doped titanium was capable of controlling the adsorption conformation of fibrinogen (a major plasma protein) acting against *P. aeruginosa* (Figure 11D).^[^ ^35^ ^]^ This study indicates that calcium‐doped materials can cooperate with the host's immune systems in acting against microbial colonization. Such materials are promising in clinical translation; however, this aspect is largely overlooked by the antimicrobial communities.

### 6.4 Other applications

Additionally, calcium is also used as an antimicrobial agent in farms, food industries, and personal hygiene. Thammakarn and colleagues fabricated nano‐sized calcium oxide (with an average diameter of 500 nm) by calcination of scallop shell powder and grinding. Solutions of nano calcium oxide could inactivate the avian influenza virus, Newcastle disease virus, and goose parvovirus within 5–30 s, which benefits veterinary medications.^[^ ^281^, ^282^, ^283^ ^]^ Similarly, calcium oxide produced by heat‐treating scallop and oyster shells at 1050°C was able to affect the permeability of fungus membranes and exhibited obvious activity against *Physalospora Agricola Nose* and *Rhizoctonia solani Kühn*, showing the material's potential as an agriculture fungicide.^[^ ^281^, ^282^, ^283^ ^]^ Moreover, the antimicrobial activity of food additive‐grade calcium hydroxide against *Salmonella Infantis* and *Salmonella Enteritidis* on eggshells was reported by Alam and colleagues. The high efficacy against foodborne bacteria supports that the material is a good candidate for the enhancement of biosecurity at farms and egg processing plants.^[^ ^284^ ^]^ The synergistic effects of food additive‐grade calcium hydroxide and quaternary ammonium compounds (common disinfectants used at livestock farms; however, their efficacy can be diminished by organic contamination or at low temperatures) were studied by Kabir et al. They found the combination of the two effectively inactivates various viruses (*f*owl adenovirus, avian reovirus, avian influenza virus, and Newcastle disease virus) and bacteria (*S. infantis* and *E. coli*) at low temperatures (minus 20°C).^[^ ^285^ ^]^ In the food industry, calcium oxide derived from the thermal treatment (1000°C for 1 h) of scallop shell powder has sporicidal action against *Bacillus subtilis*, demonstrating a safe antimicrobial for environmental preservation and food processing.^[^ ^281^, ^282^, ^283^ ^]^ Sato et al. demonstrated that the microbicidal efficacy of scallop‐shell‐derived calcium oxide was due to the high pH generated by material hydration, and under organic contaminations, this material suspension (pH 12.4) showed ten times higher bactericidal activity than hypochlorous acid (pH 6) and sodium hypochlorite (pH 8), demonstrating a potential disinfectant for environmental and food hygiene.^[^ ^286^ ^]^ The material could completely inactivate the bacterial biofilm of *Listeria innocua* (ATCC 33090, foodborne pathogens) with a 30‐minute treatment (10 mg mL^−1^, pH 12.5), showing a potentially powerful disinfection agent in the food industry.^[^ ^287^ ^]^ Choi et al. developed a calcium oxide‐based microbial decontamination assay that inhibited the growth of *Penicillium digitatum* (a fungus is predominate on physically damaged fruits) in mandarins during storage at 25°C, suggesting the material's potential for improving food storability.^[^ ^288^ ^]^ In personal hygiene, by using nano‐sized scallop‐shell derived calcium oxide (100–800 nm), Nakamura et al. produced a colorless and transparent suspension (with a pH > 12.7), which eliminated more than 99.9% of influenza A (H1N1) and Feline calicivirus, *E. coli*, *P. aeruginosa*, *Salmonella*, and *S. aureus* within 15 min. This study validated the significance of spraying disinfection of contaminated medical masks for multiple uses in personal hygiene.^[^ ^289^ ^]^ Fukuda and colleagues developed a high‐velocity steam‐air micromist jet spraying scallop‐shell derived calcium oxide suspensions for skin cleaning (or wounds) and infection prevention.^[^ ^290^ ^]^ These studies verified the antimicrobial efficacy of calcium oxide and calcium hydroxide. The action mechanism of these materials to microbes is normally explained by their capability in alkaline micro‐environment production (high pH); however, the roles of calcium ions released by the materials are unknown.

## 7 CONCLUDING REMARKS

Here, calcium's merits in ionization (small ionic radius), hydration (low hydration energy and a small hydrated ion radius), coordination (high and variable coordination numbers), and stereochemistry (variable bond length and angle) are illuminated to set a basis for understanding the material's structural former or trigger roles in proteins. Moreover, the involvement of calcium in pathogens’ integrity, motility, and metabolism maintenance is highlighted to uncover potential calcium‐associated antimicrobial targets, including calcium‐dependent bacterial envelope maintenance, calcium‐dependent metabolism homeostasis, and calcium‐dependent infection pathways. Calcium's orchestration in the immune system and recent uses in infection medications are analyzed to sketch future directions for calcium‐based antimicrobials. Overall, an integrated view of calcium in antimicrobial defense is established.

As we demonstrated, calcium is deeply involved in maintaining the integrity, motility, and metabolism of pathogens, including bacteria, viruses, and fungi. By manipulating the extracellular calcium levels, the integrity of bacterial envelopes can be disrupted, the adhesion and motility of bacterial cells can be largely reduced, the homeostasis of reactive oxygen species generation in bacteria can be interfered with, the infection pathways of virus can be blocked, and the maintenance of fungus can be disordered. Moreover, calcium is used by immune cells (neutrophils, lymphocytes, and platelets) for triggering antimicrobial ROS generation, driving extracellular trap formation, promoting granule exocytosis, and strengthening bacterial clearance capabilities. At protein levels, calcium is involved in the production and stabilization of various antimicrobial proteins and the establishment or exposure of antibacterial structures. Altogether demonstrates that control of calcium is a promising pathway leading to disinfection. However, since calcium is an essential nutrition for both mammalian and microbial cells, it is hard to boost the efficacy of the immune system or synthesize antimicrobials by using calcium while simultaneously preventing bacterial use of the material. This poses challenges for the control of calcium release or targeted delivery. That is why the direct application of calcium for infection medications is still very limited, looking forward to receiving future efforts from the antimicrobial communities. In addition, the human immune systems make sophisticated use of calcium for disinfection (as illuminated in the fifth part); the pathway for controlling calcium's antimicrobial actions or its cooperation with the immune systems in bacterial clearances is still difficult. Future works in this direction are likely to develop a new generation of calcium‐based implantable medical devices with less infection tendency but better tissue compatibility. More importantly, the exact mechanism underlying the antimicrobial efficacy of calcium is largely unclear. It is imperative to develop in situ techniques for tracking the detailed interactions of calcium with proteins, bacteria, and mammalian cells. This will facilitate a clearer understanding of the biological pathway and specific sites involved in calcium‐triggered events, which is invaluable for controlling the antimicrobial actions of calcium and developing safe disinfection assays.

In conclusion, calcium is an overlooked target in antimicrobial defense and future works on developing calcium‐based antimicrobials are promising in clinical translation.

## CONFLICT OF INTEREST STATEMENT

The authors declare no conflicts of interest.
